# A super-potent tetramerized ACE2 protein displays enhanced neutralization of SARS-CoV-2 virus infection

**DOI:** 10.1038/s41598-021-89957-z

**Published:** 2021-05-19

**Authors:** Ami Miller, Adam Leach, Jemima Thomas, Craig McAndrew, Emma Bentley, Giada Mattiuzzo, Lijo John, Ali Mirazimi, Gemma Harris, Nadisha Gamage, Stephen Carr, Hanif Ali, Rob Van Montfort, Terence Rabbitts

**Affiliations:** 1 Institute of Cancer Research, 15 Cotswold Road, Sutton, London, SM2 5NG UK; 2 National Institute for Biological Standards and Control, Blanche Lane, South Mimms, Hertfordshire, EN6 3QG UK; 3 Department of LABMED, National Veterinary Institute (SVA), 751 89 Uppsala, Sweden; 4 Karolinska Institute, 17177 Stockholm, Sweden; 5 Research Complex at Harwell, Rutherford Appleton Laboratory, Oxon, OX11 0FA UK; 6 Membrane Protein Laboratory, Diamond Light Source, Harwell Science and Innovation Campus, Didcot, OX11 0DE UK; 7 Quadrucept Limited, 1010 Cambourne Road, Cambridge, CB23 6DW UK

**Keywords:** Drug discovery, Immunology

## Abstract

Approaches are needed for therapy of the severe acute respiratory syndrome from SARS-CoV-2 coronavirus (COVID-19). Interfering with the interaction of viral antigens with the angiotensin converting enzyme 2 (ACE-2) receptor is a promising strategy by blocking the infection of the coronaviruses into human cells. We have implemented a novel protein engineering technology to produce a super-potent tetravalent form of ACE2, coupled to the human immunoglobulin γ1 Fc region, using a self-assembling, tetramerization domain from p53 protein. This high molecular weight Quad protein (ACE2-Fc-TD) retains binding to the SARS-CoV-2 receptor binding spike protein and can form a complex with the spike protein plus anti-viral antibodies. The ACE2-Fc-TD acts as a powerful decoy protein that out-performs soluble monomeric and dimeric ACE2 proteins and blocks both SARS-CoV-2 pseudovirus and SARS-CoV-2 virus infection with greatly enhanced efficacy. The ACE2 tetrameric protein complex promise to be important for development as decoy therapeutic proteins against COVID-19. In contrast to monoclonal antibodies, ACE2 decoy is unlikely to be affected by mutations in SARS-CoV-2 that are beginning to appear in variant forms. In addition, ACE2 multimeric proteins will be available as therapeutic proteins should new coronaviruses appear in the future because these are likely to interact with ACE2 receptor.

## Introduction

The cross-species propensity of coronaviruses, like SARS-CoV and now SARS-CoV-2^1,2^, has challenged our preparedness to combat diseases that suddenly appear and which spread to pandemic levels. SARS-CoV-2 is the aetiological agent of COVID-19^3^ and causes a respiratory disease with a high degree of variation in severity, leading to a large death toll world-wide. Therapeutic approaches to COVID-19 largely depend on preventing the SARS-CoV-2 coronavirus from infecting epithelial cells in airways such as in development of vaccines^4,5^ and potentially antibodies^6,7^. These can interact with the virus spike coat proteins to either cause T cell-based immunity or interference with viral binding to the ACE2 cell receptor for internalisation. The expression of the extracellular domain of ACE2^8^ and an ACE2-Fc fusion, used to study cardio-hypertension models, have shown great promise for use in COVID-19 therapies^9^. While the soluble ACE2 has rapid clearance time^10^, the addition of the Fc region renders the molecule longer lived in the blood circulation^9^.

In our current work, we have developed and characterised a new potent form of ACE2 protein which comprises four ACE2 extracellular domains by implementing a new technology that generates tetrameric proteins, termed Quads^11^. This Quad protein binds the SARS-CoV-2 spike protein receptor binding domain (RBD) with high avidity and interferes with virus infection to a greater extent than other available recombinant ACE2 proteins, producing a super-potent molecule suitable for development for therapeutic use.

## Results

### Characterization of ACE2 protein multimers

**Figure 1:**
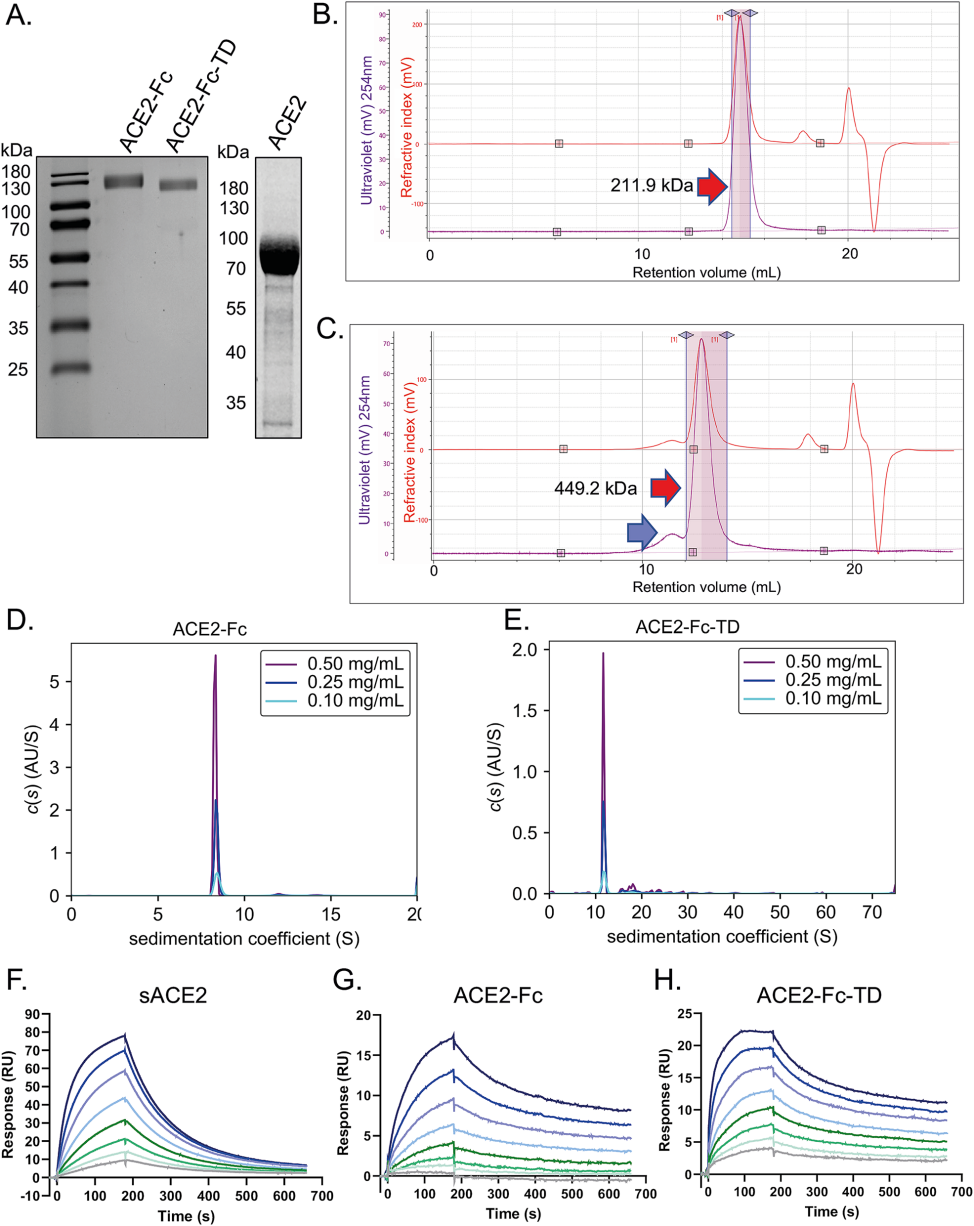
Assessment and characterization of the multimeric ACE2 proteins. The three ACE2 proteins used in this study were characterized for their molecular properties. 200 μg of ACE2-Fc, ACE2-Fc-TD or sACE2 were separated by SDS-PAGE alongside protein molecular weight markers as indicated (**A**). The molecular size of the ACE2 complexes determined by SEC–MALLS experiments. The molecular weight of the small peak in the ACE2-Fc-TD SEC–MALLS (indicated by the blue arrow, **C**) was not calculated due to low signal and likely corresponds to a mixture of higher molecular weight species observed in the AUC data (**D**,**E**). The molecular sizes were corroborated using AUC determining sedimentation coefficient distributions for ACE2-Fc and ACE2-Fc-TD (**D**,**E**). Data are shown for three samples of each protein measured at different protein concentrations. The effect of ACE2 multimerization on SARS-CoV-2 binding affinity was established using SPR (**F**–**H**). Biotinylated SARS-CoV-2-RBD was captured on a streptavidin-coated chip and ACE2 antibodies flowed over the surface at 1.56, 3.13, 6.25, 12.5, 25, 50, 100 and 200 nM. Sensograms are shown for sACE2 (**F**), ACE2-Fc (**G**) and ACE2-Fc-TD (**H**). The dissociation rates of the multimeric species ACE2-Fc and ACE2-Fc-TD are slower than for monomeric sACE2, leading to higher affinity of ACE2-Fc-TD for SARS-Cov-2-RBD. Kinetic parameters and data fits are shown in Supplementary Fig. S2. Uncropped images, showing relevant parts full length Coomassie stained SDS-PAGE gels (with indicated stained molecular weight markers), are displayed in Supplementary Fig. S4. A ChemiDoc Imaging system (BioRad) was used to capture images.

We have previously developed an antibody engineering platform that takes advantage of a human p53 domain that naturally invokes tetramerization to form tetrameric antibody formats^11^. An advantage of the p53 tetramerization domain (TD)^12^ is that it is self-assembling, naturally occurring and can be located internally or at the terminus of proteins to form tetramers. Accordingly, we have harnessed this capability to generate multimeric Quad versions of the SARS-CoV-2 cellular receptor protein ACE2. We used Expi293F cells as the expression vehicle for secretion of the extracellular domain of ACE2 (sACE2), the IgG fusion with ACE2^9^ (ACE2-IgG, herein called ACE2-Fc) and the tetrameric equivalent, ACE2-Fc-TD. These three proteins are expressed in high yield from Expi293F cells (protein samples were separated on SDS-PAGE (Fig. 1A)).

The confirmation of the respective dimeric and tetrameric structures of ACE2-Fc and ACE2-Fc-TD respectively was obtained by combining size exclusion chromatography (SEC) with multi-angle light scattering in SEC–MALLS and with analytical ultracentrifugation (AUC). Following the Ni-affinity chromatography, the proteins were further purified by SEC using Superose 6. Since each monomeric ACE2-Fc-TD protein has a p53 tetramerization domain, there is a potential for multimeric structures to form. We used SEC–MALLS to assess this possibility as this avoids the limitations of SEC that might arise with non-globular proteins. SEC–MALLS traces confirmed the ACE2-Fc protein elutes as a single peak and light scattering analysis shows the molecular weight of the sample within the peak to be 211 kDa (Fig. 1B), consistent with a dimeric species in solution, as predicted from the original description of the ACE-Fc/ACE2-IgG^9^. The main peak of ACE2-Fc-TD Quad protein corresponds with a molecular weight of 449 kDa, consistent with a tetramer of ACE2 domains (see below). The sample also contains a small peak of higher molecular weight species (Fig. 1C) that presumably occur through combinations tetramerization via P53 TD and dimerization via the immunoglobulin domain. The SEC–MALLS data are summarized in Supplementary Table S1A.

The molecular mass of ACE2-Fc and ACE2-Fc-TD were confirmed by AUC analysis (data are summarized in Supplementary Table S1B). The ACE2-Fc sample sedimented predominantly as a single species (Fig. 1D) with an average molecular weight of 222 kDa (Supplementary Table S1B), again consistent with a dimeric species in solution. The ACE2-Fc-TD construct sedimented predominantly as a species with an average molecular weight of 407 kDa (Fig. 1E), consistent with a tetramer, although small amounts of higher molecular weight species were also observed. Analysis of the overall shape of the protein complexes was attempted using size-exclusion chromatography with small-angle X-ray scattering (SEC-SAXS). This technique is advantageous for samples containing multiple species, since the size-exclusion chromatography step allows their separation enabling scattering curves to be collected for each individual protein. Kratky plots revealed ACE2-Fc to be globular with moderate flexibility and the molecular envelope calculated for ACE2-Fc consisted of three lobes, one for the IgG1 Fc region and two for the ACE2 extracellular domains. A representative structural model, whereby the ACE2 extracellular domain is fused to the IgG dimerization domain fits well into the envelope (Supplementary Fig. S1). The two larger lobes are occupied by ACE2 extracellular domain with the Fc dimerization domain located in the third. The larger size of the lobes relative to the model is a result of the flexibility of the protein, since the antibody-derived linkers between the Fc and ACE2 domains allow their rotation relative to each other.

**Figure 2:**
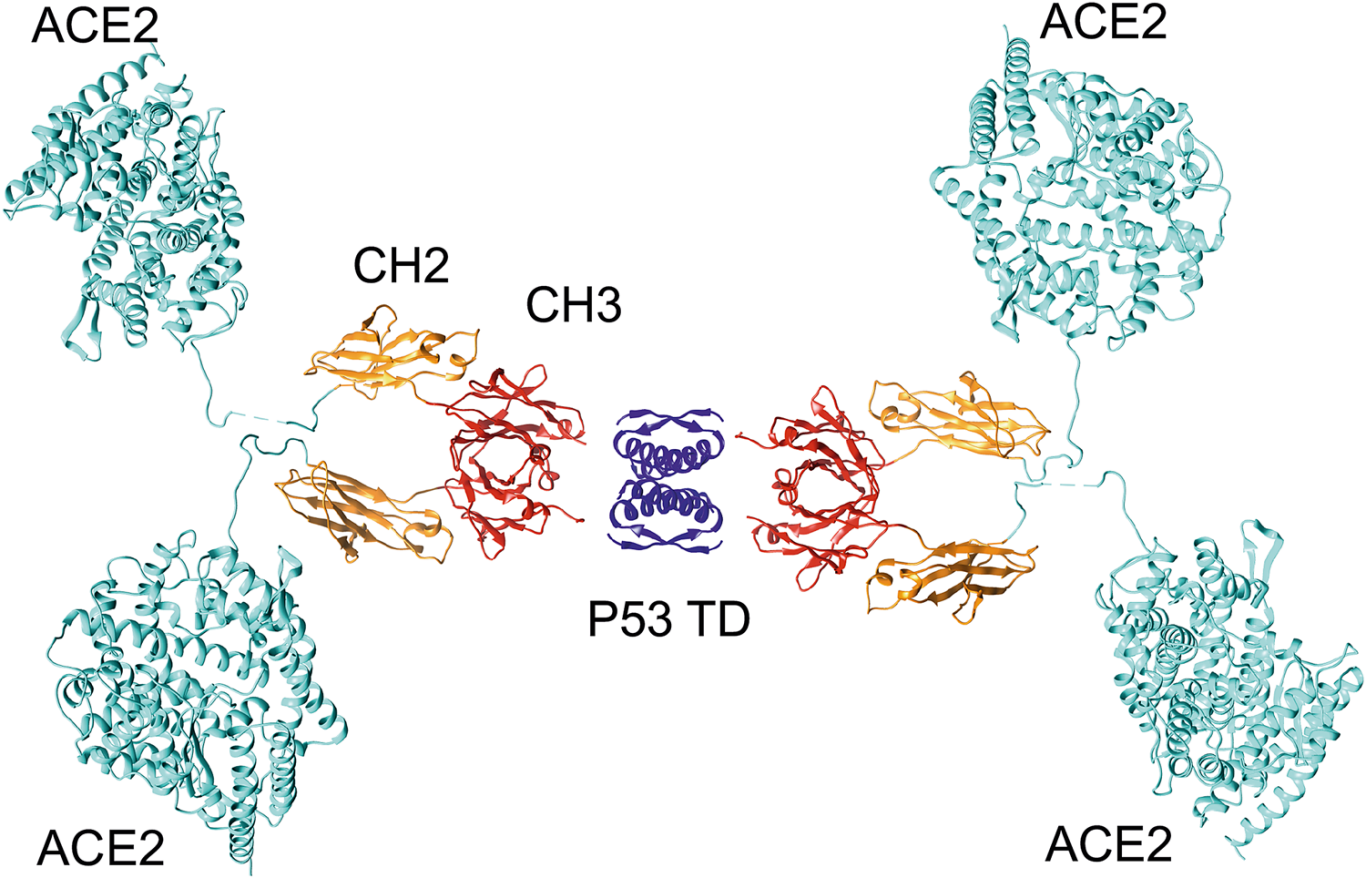
Molecular model of the ACE2-Fc-TD Quad protein. A model of the putative structure of the ACE2-Fc-TD Quad protein, assuming the P53 tetramerisation domain is the major driver of oligomerisation. SEC-SAXS, SEC–MALLS and AUC data support an elongated and tetrameric structure for this protein. The colour scheme comprises the ACE2 extracellular domain coloured in cyan, the IgG CH2 domain in orange and the IgG CH3 domain in red, with the tetramerization domain of P53 (P53 TD) coloured blue. A gap has been left between P53 TD and CH3 domain of each IgG to account for the flexible 6 amino acid linkers between these domains.

Unfortunately, we were unable to obtain a similar envelope for the ACE2-Fc-TD protein due to a higher degree of flexibility within the larger protein that prevented the calculation of the molecular envelope. Nevertheless, we were able to generate a model of the tetrameric form that the ACE2-Fc-TD takes based on the SEC–MALLS and AUC data is depicted in Fig. 2. The model has a theoretical Rg in good agreement with that measured in SEC-SAXS experiments and suggests that the Quad ACE2-Fc-TD has tetrameric valency for ACE2 domains. Such a structure is reminiscent of the organisation of immunoglobulin IgA, where two immunoglobulins are held together by the J-chain through disulphide bonds.

### The ACE2 tetramer captures SARS-CoV-2 spike protein

The ACE2-Fc protein was shown previously to bind the SARS-CoV and SARS-CoV-2 viral spike RBD and to neutralize viruses pseudotyped for the respective spike proteins of both SARS-CoV and SARS-CoV-2^9^. The ability of the tetrameric ACE2-Fc-TD to interact with the SARS-CoV-2 viral spike protein compared to ACE2-Fc and to sACE2 was studied using surface plasmon resonance (SPR). ACE2-Fc and ACE2-Fc-TD were found to have slower dissociation rates than sACE2 (k_off_ 0.0019, 0.0017 and 0.012 s^-1^ respectively). This resulted in increased affinity of ACE2-Fc-TD for SARS-Cov-2-RBD compared to that of sACE2, with K_d_ values 3.9 nM and 22 nM respectively (Fig. 1F–H and Supplementary Fig. S2).

**Figure 3:**
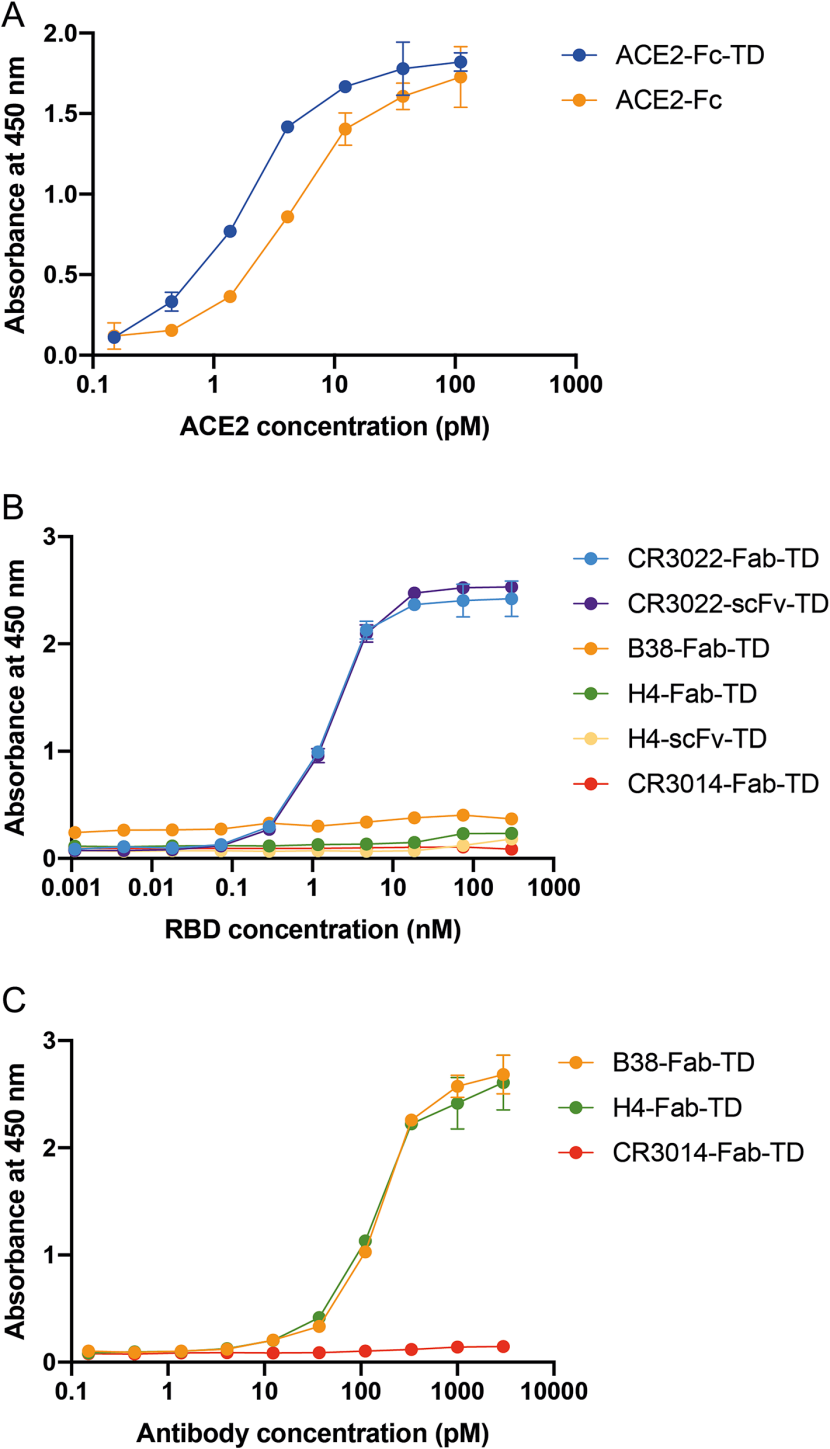
ELISAs with ACE2 protein binding to SARS-CoV-2 spike protein. The ability of ACE2 Quads to bind to SARS-Cov-2 virus spike protein was analysed by binding assays with and without anti-SARS-Cov-2 tetramers. (**A**) Direct ELISAs of ACE2 Binding to SARS-Cov2-RBD. Increasing amounts of ACE2 proteins were added to SARS-CoV-2 RBD protein coated ELISA plates followed by detection with 100ul of anti- IgG-HRP and, after washing, TMB was added for 20 min and the absorbance read at 450 nm. The ACE2-Fc-TD EC_50_ ~ 2 pM and ACE2-Fc EC_50_ ~ 6 pM, representative of two experiments. (**B**) Sandwich ELISA detection of anti-SARS-CoV-2 spike protein. ACE2-IgG-TD coated ELISA wells (300 ng/well as the capture protein) were incubated with SARS-CoV-2 RBD in the range of 0-300 nM in PBS containing 0.05% Tween-20 overnight at 4 °C. Anti-RBD antibodies were added at 1 nM in PBS containing 0.05% Tween-20 and incubated for 3 h at room temperature and bound antibody detected with anti-His-HRP and, after washing, addition of TMB and the absorbance read at 450 nm. (**C**) Direct ELISAs of anti-CoV-2 spike protein binding antibodies. ELISA wells were coated with 200 ng SARS-CoV-2 RBD protein 200 ng/well) in PBS, pH 7.4 and detected with the range of anti-CoV-2 antibodies. The bound antibody was detected with an HRP-conjugated anti-His antibody followed by addition of TMB and determining the absorbance read at 450 nm.

Confirmation that the ACE2-Fc-TD retains potent binding to SARS-CoV-2 viral spike RBD protein was obtained using direct enzyme-linked immunosorbent assays (ELISAs) in which SARS-CoV2 RBD protein was coated on plates and the binding of various ACE2-Fc-TD or ACE2-Fc concentrations assessed. The binding affinity of both proteins was similar, yielding ACE2-Fc-TD EC_50_ 2 pM and ACE2-Fc EC_50_ 6 pM (Fig. 3A). Having established that both engineered ACE2 proteins effectively bind SARS-CoV2 RBD, sandwich ELISAs were performed with ACE2-Fc-TD coated plates to capture SARS-CoV2 RBD and detected with anti-SARS-CoV-2 antibodies also made in the tetrameric form, either as single chain variable fragment (scFv) or immunoglobulin Fab fragments. The sequences of the tetramer antibody fragments used are given in Supplementary Fig. S3 and were based on published data for CR3022 and CR3014^13^, and H4 and B38^14^. Our data show that RBD captured by ACE2-Fc-TD is detected by CR3022 Fab-TD and the CR3022 scFv-TD but not by CR3014-Fab-TD which is known to be a non-binder^15^ and serves as the negative control in our experiments (Fig. 3B). The H4 and B38 antibodies have been reported to neutralize SARS-CoV-2 virus infection with distinct epitopes on the spike protein^14^. None of our engineered tetrameric Quad antibodies, H4-scFv-TD, H4-Fab-TD or the B38 Fab-TD, had any binding to ACE2 captured RBD. However, direct ELISA using H4-Fab-TD, H4-scFv-TD or B38 Fab-TD-coated plates could detect SARS-CoV2 RBD (using CR3014-Fab-TD as negative control) (Fig. 3C). The binding sites for H4 and B38 antibodies must therefore overlap with ACE2 receptor epitopes on the SARS-CoV2 RBD. Our tetramerized anti-SARS-CoV-2 antibodies bind to the spike protein, as do the original IgG antibodies.

### The ACE2 tetramer displays enhanced interference in SARS-CoV-2 infection

**Figure 4:**
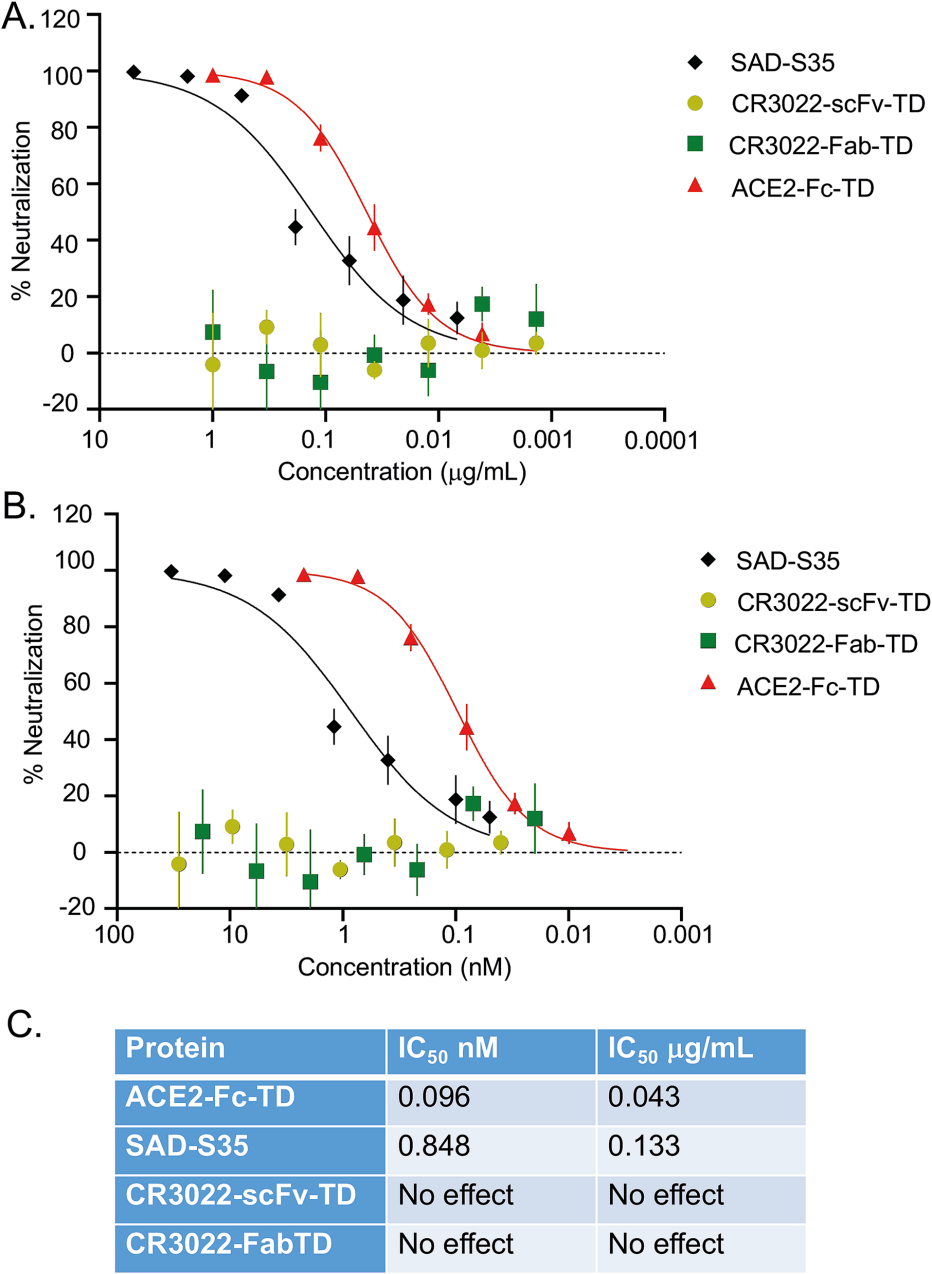
Tetrameric ACE2-Fc-TD is a potent inhibitor of virus infection. The viral infection neutralizing potency of tetrameric ACE2-Fc-TD Quad was examined in SARS-CoV-2 pseudovirus infection assays. Lentiviral pseudotyped virus expressing the SARS-CoV-2 spike protein was incubated for 1 h with SAD-S35 (AcroBiosystems) and ACE2-Fc-TD alongside either tetrameric scFv or Fab variants of CR3022. HEK 293 T/17 cells expressing ACE-2 and TMPRSS2 were subsequently incubated with the mixtures and luciferase activity detected after 60 h. Graphs represent the percentage neutralization plotted as μg/mL (**A**) or molarity (**B**) calculated by non-linear regression analysis. Data are represented as mean ± SEM. IC_50_ values shown in (**C**) were calculated using GraphPad Prism software.

The binding data for ACE2-Fc-TD demonstrate that this engineered protein binds with high affinity to the SARS-CoV-2 viral spike protein. A comparison of the efficacy of the ACE2 proteins to act as decoy proteins in neutralization of SARS-CoV-2 viral infection was carried out with pseudotyped virus assays (Fig. 4A,B). We used SARS-CoV-2 pseudotyped lentivirus transduction of HEK293T cells expressing the ACE2 receptor and TMPRSS2 (T2) to compare neutralization by ACE2-Fc-TD (tetrameric for ACE2 extracellular domains) with a neutralizing anti-CoV-2 monoclonal antibody (SAD-S35) that binds to the CoV-2 spike protein. In this assay, a pseudotype-associated reporter *luciferase* gene is used to measure levels of pseudovirus uptake by ACE2/T2-expressing recipient cells. Both ACE2-Fc-TD and SAD-S35 antibody interfere with viral infection whereas the previously reported CoV-2 spike antibody (CR3022, which binds to the spike protein but does not neutralize^13^) does not, either as Quad tetrameric scFv or Fab (Fig. 4A,B). The IC_50_ values determined for ACE2-Fc-TD are 0.096 nM and 0.848 nM for SAD-S35 (Fig. 4C) demonstrating that ACE2-Fc-TD potency is around tenfold higher than the antibody.

**Figure 5:**
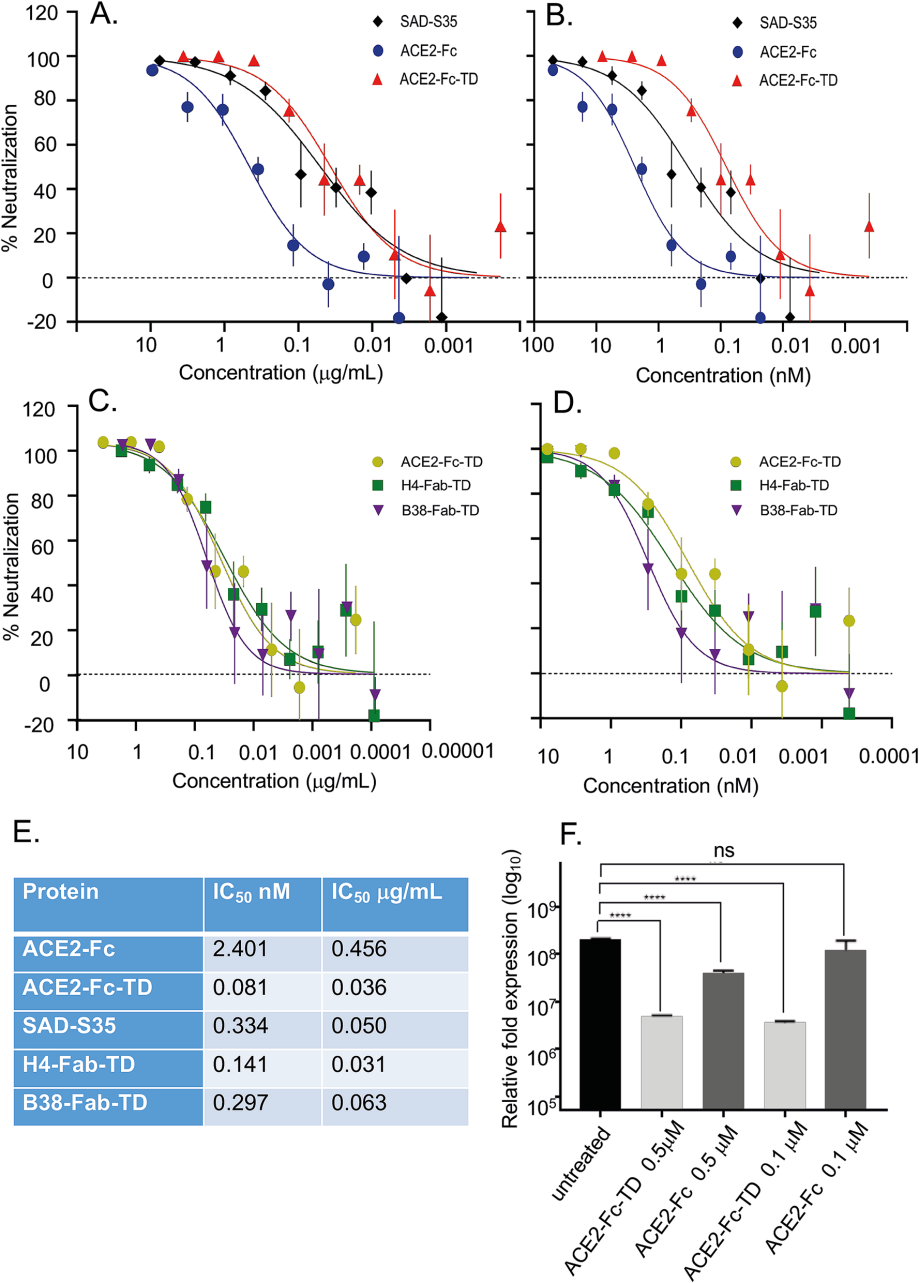
Tetrameric ACE2-Fc-TD out competes dimeric ACE2-Fc in viral neutralization assays. The neutralizing potency of tetrameric ACE2-Fc-TD Quad in inhibition of viral infection was examined in pseudovirus and live SARS-CoV-2 infection assays. (**A**,**B**) Lentiviral pseudotyped virus expressing SARS-CoV-2 spike protein were combined with SAD-S35 (AcroBiosystems), with ACE2-Fc, or with ACE2-Fc-TD and added to HEK 293 T/17 cells expressing ACE-2 and TMPRSS2 after 1 h. The cells were incubated for 60 h before luciferase activity, indicative of viral entry, was determined. (**C**,**D**) Further compares the neutralization of ACE2-Fc-TD, with H4-Fab-TD or with B38-Fab-TD. IC_50_ data (**E**) were calculated using GraphPad Prism software and compare the efficacy of the two forms of soluble ACE2 proteins with the commercially available anti-SARS-CoV-2 antibody SAD-S35 and tetramerized anti-SARS-CoV-2 Fab antibodies H4 and B38^14^. (**F**) ACE2-Fc (dimeric for ACE2) and ACE2-Fc-TD (tetrameric for ACE2) proteins were preincubated with SARS-CoV-2 for 30 min at 37 °C. Vero E6 cells were infected with the ACE2-virus mixtures for 1 h at 37 °C before washing and continued incubation for 15 h at 37 °C. Cells were recovered and viral RNA was assayed by qRT-PCR. The graph shows relative fold change of viral RNA based on the Ct values compared to cellular RNA levels of mock infected cells. Data are represented as mean ± SD. (Student’s t test: ****P < 0.0001 p * < 0.05).

We also compared the potency of the ACE2-Fc-TD Quad protein with the original ACE2-Fc protein in the ability to neutralize the pseudovirus infection (Fig. 5A,B). The IC_50_values are 2.585 nM and 0.113 nM for ACE2-Fc and ACE2-Fc-TD respectively (panel E), meaning that the Quad protein is more than 20 times more potent in viral neutralization. Finally, using a pseudovirus infection assay, the efficacy of ACE2-Fc-TD was compared with the neutralization power of two known neutralizing antibodies, H4 and B38^14^ that we have made into Fab tetramer versions and showed they could bind to the SARS-CoV-2 spike protein in ELISA (Fig. 3). The ACE2-Fc-TD protein out-performs both of these antibodies in the viral neutralization assays (Fig. 5C,D) with an IC_50_ at 0.081 nM compared with IC_50_ 0.141 nM and 0.297 nM for H4-Fab-TD and B38-Fab-TD, respectively (Fig. 5E).

These assays were performed with a non-replicative pseudotyped lentivirus carrying only the SARS-CoV-2 spike protein. We therefore wished to assess the effect of ACE2 valency on replication by infectivity using a previously described live SARS-CoV-2 virus isolate^8^. We tested the potential of dimeric ACE2 (ACE2-Fc) and tetrameric ACE2 (ACE2-Fc-TD) to interfere with replicative infection by this SARSCoV-2. Vero-E6 cells were infected with SARS-CoV-2 and monitored for viral replication by qRT-PCR^8^. Consistent with the data obtained with the pseudotyped lentivirus, infection of cells is lowered about 10 times in the presence of tetrameric ACE2-Fc-TD decoy compared to the dimeric ACE2 (Fig. 5F). ACE2-Fc-TD is a functional decoy protein even at nanomolar concentration. Our results show that tetrameric ACE2 could efficiently inhibit SARSCoV-2 attachment to the cells, thereby reducing infection, and that valency of the ACE2 protein is a key determinant of increased potency*.*

## Discussion

We have compared the efficacy of monomeric (sACE2), dimeric (ACE2-Fc) and tetrameric (ACE2-Fc-TD) ACE2 proteins in binding to SARS-CoV-2 spike protein antigens and in the interference of virus binding and infection via the cell surface receptor ACE2. Increasing the valency of ACE2 proteins correlates well with increased potency. Soluble ACE2 extracellular domain proteins show efficacy for inhibition of virus infection^8^. In addition, the engineered dimeric ACE2-Fc has been shown to interfere with virus infection^9^. Our work on self-assembling antibodies showed that increasing valency enhances the functional affinity^11^ and we have applied this method to increasing the valency of ACE2. We have carefully established the molecular characteristics of the Quad protein ACE2-Fc-TD protein showing that the tetravalency of the ACE2 domains is displayed to facilitate potency increase and is the most efficacious of the proteins tested.

Recently, new formats for ACE2 have been described that either occur as antibody-ACE2 fusion proteins ACE2^16^, a new ACE2 de novo design that includes three tandem ACE2 repeats^17^ or a mutant, engineered ACE2^18^. All these novel proteins can inhibit viral infection. The Quad version of ACE2 described here is made from natural ACE2 sequences using a simple protein engineering technology^11^ that does not introduce potential immunogenicity. The Quad version of ACE2 could be developed into a therapeutic decoy agent, potentially valuable in testing for SARS-CoV-2 infection, in diagnosis of COVID-19 and as a therapeutic molecule that could potentially be used intra-nasally or systemically. Further, unlike antibodies targeting SARS-CoV-2 RBD, using natural ACE2 sequences means that it will be unlikely to be affected by any mutations that may occur in the coronavirus^19^, since ACE2 is likely to remain as the receptor target for new mutants of the virus. Yields in excess of 60 mg per litre of culture were achieved in the Expi293 culture system, with only limited optimization thus far, so that gram quantities are feasible for manufacture. Vaccination strategies for COVID-19 prevention^5^ will ultimately prove to greatly mitigate the threat from SARS-CoV-2 in the population. However, therapeutic molecules acting directly on SARS-CoV-2 infection are still urgently needed. While therapeutic antibodies that bind to the spike protein may interfere with the interaction of the virus with the cell-surface receptor ACE2, non-cell bound forms of ACE2 could themselves be developed as therapeutics because they act in SARS-CoV-2 viral defense by mimicking receptor as a decoy protein. They should be able to titrate viral particle receptor binding spike proteins and valency will, in that case, be important because the protein can be administered at a lower dose. It is possible that higher order versions of ACE2 can be engineered by using the tetramerization domain strategy and these could further improve potency. There is already evidence detailing ACE2 decoys are effective in clinical trials, such as a recombinant human ACE2 is in phase II for treating COVID-19 patients and the data show, it is well tolerated with good safety profile (NCT04335136). An important feature of the super-potent ACE2 Quad is that it gives an enhancement over natural ACE2 sequences and therefore should be a safe and efficacious approach to developing more potent therapeutics. In addition, the tetramers can be building blocks for yet more potent ACE2 molecules. In addition, since soluble ACE2 has a short in vivo residence^10^, the creation of high molecular weight tetramers should facilitate long circulation times. Moreover, the tetramerization technology^11^ is full flexible and easily adapted to any new proteins that may be devised. The method involves simple protein engineering and allows production of secreted multimeric proteins with high yields.

Aside from possible use in other clinical indications, such as cardiac hypertension^20,21^, where the ACE2 Quad proteins will be effective, these enhanced tetramerized ACE2 Quad complexes have wider therapeutic applications. Should any future new SARS-related coronaviruses emerge in the human population, these will most likely enter cells by the same ACE2 receptor mechanism and the availability of characterised, enhanced potency decoys could be immediately available to avert future pandemic situations. This applies to any pathological virus using ACE2 for cell entry. The implementation of potent human ACE2-derivatives is unlikely to display immunogenic properties per se*.* Recent data have shown the occurrence of viral spike protein mutations^22–24^ and some shown to affect binding of anti-SARS-CoV-2 antibodies^19^ that are themselves in clinical trials. Given the need for ACE2 binding for viral infection into cells, any coronaviruses mutations that develop will be unlikely to evade ACE2 binding. The ACE2-decoy strategy could negate the requirement for developing complex antibody cocktails and thus ACE2-decoy Quads could potentially be developed as a standalone efficacious therapeutic.

## Methods

### Cloning, expression and purification of SARS-CoV-2 receptor binding domain

The coding sequence of the SARS-CoV-2 spike protein was amplified by PCR using a template kindly provided by Dr. Peter Cherepanov. The PCR product, together with two gene fragments encoding the GP67 leader sequence and a C-terminal Avitag-HRV3C-His8 tag, were assembled using NEBuilder HiFi (NEB) into the p10 promoter of a pFBDM baculovirus expression vector that had been digested with XmaI & KpnI.

Baculovirus were generated according to Bac-to-Bac protocols (Invitrogen) and used to infect 800 mL of High Five™ cells grown in shaker cultures at 27 °C with Sf-900™ III SFM. After 5 days the culture media was collected, clarified by centrifugation (30 min, 10,543×*g*) and concentrated over eightfold using a Vivaflow 200 Crossflow Cassette (Sartorius). HEPES pH7.4, NaCl & glycerol were added to give final concentrations of 50 mM, 150 mM and 5% respectively, prior to freezing.

The protein was purified by nickel affinity purification. 100 mL of concentrated media was thawed, filtered through 1.2 μm syringe filters and applied to a 5 mL HisTrap excel. The column was sequentially washed with 50 mL 4% buffer B (20 mM Tris pH8.0, 150 mM NaCl, 500 mM imidazole), 17 mL 8% buffer B & 17 mL 16% buffer B, prior to elution with 17 mL 100% buffer B. Eluted protein was incubated with HRV3C protease (19U/mg substrate), BirA (1 μg/nmole substrate), biotin (10 x [substrate]), ATP (1 mM) & MgCl2 (3.3 mM) for 5 h prior to loading onto a HiLoad 26/600 Superdex 75 pg column (running buffer 20 mM Tris pH8.0, 150 mM NaCl) with a 1 mL GSTrap FF column in series. Peak fractions were pooled and reapplied to the 5 mL HisTrap™ Excel column. Flow through fractions were pooled, concentrated & frozen.

All columns were from Cytiva life sciences (formerly GE Healthcare).

### Cloning, expression and purification of SARS CoV-2 S1 spike protein

The PCR product of the SARS-CoV-2 spike S1 protein was cloned into pTT5 for expression in Expi293F cells. Cells were seeded to 1.5 × 10^6^/mL and cultured overnight and transfected. 60 μg of pTT5-CoV-2 RBD plasmid DNA was added to 3 mL of Opti-Mem (Thermofisher). and 160ul of ExpiFectamine (Thermofisher) added to 3 mL of Opti-Mem and both incubated for 5 min at room temperature. These were mixed and further incubated at room temperature for 30 min before adding the DNA-reagent complex to the Expi293 cells and incubating at 37 °C, 5% CO_2_ with continuous mixing at 125 rpm. 300ul of enhancer 1 and 3 mL of enhancer 2 solutions were added 16–18 h post transfection and cells incubated the 37 °C, 5% CO_2_ for a further 5 days with mixing at 125 rpm. Purification was carried as described for the SARS-CoV2-RBD.

### Cloning and expression of recombinant ACE2 and anti-CoV2 antibody fragments

The various constructs for ACE2 proteins (ACE2-Avi-HIS; ACE2-Fc; ACE2-Fc-TD) and anti-SARS-CoV-2 antibody fragments in the Quad format (CR3022-Fab-TD, CR3022-scFv-TD, B38-Fab-TD, H4Fab-TD, H4-scFv-TD, CR3014-Fab-TD) were cloned and expressed in pTT5^25^. The sequences of the coding regions of each are shown in Supplementary Fig. S3.

All proteins produced by secretion from Expi293F cells (Thermofisher) as described for the SARS-CoV-2 spike S1 protein. For the anti-SARS-CoV-2 Quad antibodies, cell supernatants were purified by nickel affinity chromatography and SEC. Approximately 60 mL of filtered culture media was applied to a 5 mL HiTrap QFF column. Following a 25 mL wash with 20 mM Tris pH8.0, 100 mM NaCl, a gradient was developed from 0 to 50% 20 mM Tris pH8.0, 150 mM NaCl, 500 mM imidazole over 100 mL.

ACE2 proteins were purified using HiTrap QFF column as for the antibodies and the peak fractions were pooled, concentrated and applied to a HiLoad 16/600 Superose 6 prep grade column in running buffer 1 × PBS (Oxoid PBS Tablets, Thermo Scientific). Peak fractions were pooled & concentrated.

### SEC–MALLS

SEC–MALLS experiments were performed using a Superose 6 10/300 Increase column (GE Healthcare) and an OMNISEC SEC–MALLS system (Malvern). The samples (100 μL, 1 mg/mL) were loaded onto the gel filtration column pre-equilibrated with PBS and eluted with one column volume (24 mL) of buffer, at a flow rate of 0.5 mL/min. Data were analysed using the OmniSEC software (v10.41, www.malvernpanalytical.com), using a refractive increment value of 0.185 mL/g.

### Analytical ultracentrifugation

Sedimentation velocity experiments were performed at 40,000 rpm, using a Beckman Optima analytical ultracentrifuge (AUC) with an An-50Ti rotor at 20˚C. Data were recorded using the absorbance (at 280 nm) optical detection system. The density and viscosity of the buffer (PBS) was measured experimentally using a DMA 5000 M densitometer equipped with a Lovis 2000ME viscometer module. The partial specific volume of the protein was calculated using SEDFIT from the amino acid sequence. Data were processed using SEDFIT, fitting to the c(s) model. Figures were made using GUSSI software (v1.4.2, https://doi.org/10.1016/bs.mie.2015.05.001).

### SEC-SAXS

All SAXS data were collected at beamline B21 (Diamond Light Source, UK) operating in SEC-SAXS mode and using an EIGER 4 M detector. Prior to data collection all protein samples were centrifuged at 15,000×*g* for 5 min to remove any insoluble aggregates. SEC-SAXS was performed using a Superose 6 10/300 Increase column (GE Healthcare) pre-equilibrated with PBS + 2% sucrose, where sucrose is added to minimise radiation damage during data collection. Protein samples (45 μL, 5 mg/mL) were injected onto the column and eluted with one column volume (24 mL) of buffer, at a flow rate of 0.5 mL/min. Scattering curves were collected throughout the elution and analysed using the SCÅTTER IV software suite (www.bioisis.net). Molecular envelopes were calculated using DAMMIN^26^ with envelopes representing the average of 13 independent runs. Representative models for ACE2-gG and ACE2-IgG-TD were manually constructed in COOT^27^ using PDB files 1R42 (ACE2), 1HZH (IgG) and 1C26 (P53) and fitted to the molecular envelope using Chimera^28^.

### Surface plasmon resonance of ACE2 proteins binding SARS-CoV-2 RBD

SPR experiments were carried out using Biacore T100 (Cytiva, formerly GE Healthcare) in PBS buffer, pH 7.4 containing 0.05% Tween-20. A streptavidin-coated SA chip (Cytiva) was washed with 3 × 30 s injections of 1 M NaCl/50 mM NaOH at 10µL/min before biotinylated SARS-CoV-2-RBD (0.2 µg/mL) was captured to target immobilisation level 1000RU. ACE2 protein binding to SARS-CoV-2-RBD was analysed at 25ºC using multi-cycle injections of sACE2, ACE2-Fc and ACE2-Fc-TD at 8 concentrations between 1.56 and 200 nM at 30µL/min for 180 s, followed by 480 s dissociation time. The surface was regenerated after each antibody injection using 1 M NaCl/50 mM NaOH at 30µL/min for 20 s contact time followed by 120 s stabilisation time. Flow cell 1 was used as a reference and so did not contain any captured SARS-CoV-2-RBD and was subtracted from all sensograms before analysis. Data were fitted to a 1:1 kinetics model using Biacore T200 Evaluation Software version 2.0 (www.cyvitalifesciences.com).

### ELISA

All ELISAs were carried out in triplicate wells. Direct ELISAs with ACE2 proteins were carried out with SARS-CoV-2 spike RBD-coated ELISA plates and 100 μL of SARS-CoV-2 RBD protein (2 μg/mL in PBS, pH 7.4) was added to each well and incubated overnight at 4 °C, along with a control well with 100ul of PBS. The wells were washed three times with PBS containing 0.05% Tween-20 and blocked with 150 μL 5% BSA in PBS by incubating for 4 h at room temperature. Excess BSA was removed by washing twice with PBS containing 0.05% Tween-20. A range of ACE2 samples 0-3000 pM in PBS containing 0.05% Tween-20 were made and 100 μL added to each well and incubated at 4 °C overnight. The plates were washed again four times with PBS containing 0.05% Tween-20 before addition of 100 μL of detection antibody (anti-IgG-horse radish peroxidase (HRP, Cell Signalling) diluted in 1: 4000 1% BSA in PBS to each well and incubate for 2 h at room temperature. The wells were washed four times with PBS containing 0.05% Tween-20 followed by the addition of 25 μL tetramethyl benzidine (TMB; ThermoFisher) and incubate for 20 min at room temperature. Reactions were stopped by addition 25 μL of 3 M HCl and absorbance read at 450 nm.

Direct ELISAs of anti-SARS-CoV2 spike protein binding antibodies. ELISA wells were coated with 200 ng/well SARS-CoV-2 RBD protein and detection with anti-SARS-CoV-2 RBD antibodies added in PBS containing 0.05% Tween-20 (range 0-3000 pM). Development and signal detection as for the ACE2 direct ELISA except using an HRP-conjugated anti-His antibody.

Sandwich ELISA detection of anti-SARS-CoV-2 spike protein. ACE2-IgG-TD coated ELISA wells (300 ng/well as the capture protein in 100 μL) were prepared by adding ACE2-IgG-TD protein in PBS, pH 7.4 to each well and incubating at 4 °C overnight. After washing the wells three times with PBS containing 0.05% Tween-20 and blocking with 150 μL 5% BSA in PBS by incubating for 4 h at room temperature and rewashing with PBS containing 0.05% Tween-20. Diluted SARS-CoV-2 RBD samples in PBS containing 0.05% Tween-20 (range 0-300 nM) were added in fourfold serial dilutions and incubated overnight at 4 °C. The captured RBD was detected using anti-spike antibodies at 1 nM in PBS containing 0.05% Tween-20 and incubating for 3 h at room temperature. Following four washes with PBS containing 0.05% Tween-20, bound antibody was detected by adding to each well 100 μL of anti-His-HRP antibody diluted in 1% BSA in PBS (1:4000) and incubating for 2 h at room temperature. The wells were finally washed four times with PBS containing 0.05% Tween-20, 25 μL of TMB was added, incubated for 20 min at room temperature, the reaction stopped by adding 25 μL of 3 M HCl and reading the absorbance at 450 nm.

### SARS-CoV-2 lentiviral pseudotyped virus neutralisation assay

SARS-CoV-2 pseudotyped lentivirus was produced by a three-plasmid transfection of HEK293T/17 cells following a similar approach to that previously described^29^. Briefly, 24 h prior to transfection cells were seeded within a 10 cm dish before transfecting with plasmids encoding the HIV-1 *gag-pol* genes (p8.91)^30^, a firefly luciferase reporter gene (pCSFLW) and the SARS-CoV-2 Spike gene (pCAGGS SARS-CoV-2-Spike) at a ratio of 1:1.5:1 ug using Fugene-HD (Promega) transfection reagent. Following overnight incubation, culture media was replenished. Supernatant was harvested at 48 and 72 h post-transfection, and stored at -80 °C. Neutralizing activity was measured by preparing two-fold serial dilutions of antibodies and incubating with 200 TCID50 of SARS-CoV-2 lentiviral pseudotyped virus for 1 h at 37 °C. HEK293T/17 cells stably expressing ACE-2 and TMPRSS2 were used as target cells, seeding 20,000 cells/well within a 96-well plate and incubating for at least 2 h at 37 oC, 5% CO_2_ before use. Following incubation, the antibody and pseudotyped virus mix was transferred to the target cells and incubated for a further 60 h at 37 oC, 5% CO_2_. Results were acquired by detection of luciferase expression using the Promega Bright-Glo assay system and GloMax Navigator plate reader, following manufacturer’s instructions. Data were normalised to untreated, transduced cells and uninfected cells as controls to determine percentage neutralisation and non-linear regression analysis performed within GraphPad Prism, interpolating IC_50_ values.

### SARS-CoV-2 virus and cell lines

SARS-CoV-2 strain used for this study was originally isolated as described previously^8^. Virus was propagated and titrated in African Green monkey cell line Vero-E6 (ATCC CRL-1586) by tissue culture infectious doses per milliliter (TCID50) method as described^31^. Vero-E6 cells were maintained in Dulbecco’s Modified Eagle’s Medium (DMEM, Thermofisher) supplemented with 1% Non-Essential Amino-Acid (Thermofisher), 10 mM HEPES (Thermofisher) and 10% FBS at 37C, 5% CO2.

### SARS-CoV-2 infection of Vero E6 cells and quantification of viral RNA

The protocols for SARS-CoV-2 virus neutralization are based on a previous publication^8^. Before cell infections, ACE2-Fc (dimeric for ACE2) or ACE2-Fc-TD (tetrameric for ACE2) purified proteins were mixed with SARS-CoV-2 virus (for multiplicity of infection of one) and incubated at 37 °C for 1 h. Vero E6 cells were previously seeded into 48-well plates at a density of (5 × 10^4^ cells per well) and after 24 h, cells were either mock infected or infected with SARS-CoV-2/ACE2 protein mixtures. The cells were incubated for 15 h at 37 °C before harvesting for quantitative real time polymerase reaction (qRT-PCR) analysis. Total cell RNA was extracted using Direct-zol RNA MiniPrep kit (Zymo Research) and the qRT-PCR was performed using the primers and probe specific for SARS-CoV-2 E-gene.

- Forward primer: 5′-ACAGGTACGTTAATAGTTAATAGCGT-3′.
- Reverse primer: 5′-ATATTGCAGCAGTACGCACACA-3′.
- Probe: ACACTAGCCATCCTTACTGCGCTTCG.

Intracellular viral RNA levels were normalized to 18S rRNA using the Taqman probe (Thermo Fisher Hs03003631-g1).

## Supplementary Information

Supplementary Information.
