# Supplementary material for: A super-potent tetramerized ACE2 protein displays enhanced neutralization of SARS-CoV-2 virus infection
Source: Sci Rep. 2021 May 19;11:10617. doi: 10.1038/s41598-021-89957-z (PMC8134500; doi:10.1038/s41598-021-89957-z)
Supplement: Supplementary file 1 — Supplementary Information. [file 41598_2021_89957_MOESM1_ESM.pdf]

A super-potent tetramerized ACE2 protein displays enhanced neutralization of SARS-CoV-2 virus infection

Ami Miller, Adam Leach, Jemima Thomas, Craig McAndrew, Emma Bentley, Giada Mattiuzzo, Lijo John, Ali Mirazimi, Gemma Harris, Nadisha Gamage, Stephen Carr, Hanif Ali, Rob Van Montfort and Terence Rabbitts

Supplementary Figure 1

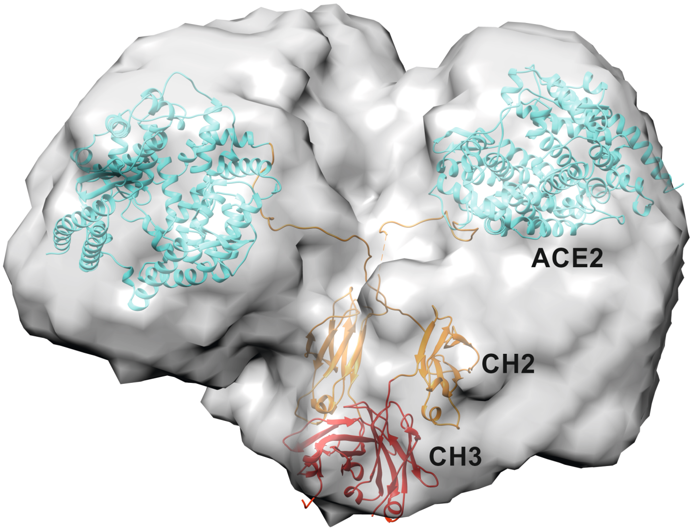

Supplementary Figure 1. Structural analysis of ACE2-Fc protein

Representative model of chimaeric molecule ACE2-IgG fitted into the experimentally determined scattering envelope (represented in grey) determined by SEC-SAXS. A structural model of the components were fitted into the envelope (using UCSF chimera; J Comput Chem doi: 10.1002/jcc.20084) and comprise the ACE2 extracellular domain coloured in cyan, the IgG CH2 domain in orange and the IgG CH3 domain in red.

Supplementary Table 1: Biophysical determination of molecular weights for ACE2-Fc and ACE2-Fc-TD

A. Molecular weights determined by SEC-MALLS

Sample	Monomeric MW (kDa)	Concentration (mg/mL)	Mn (kDa)	Mw (kDa)	Mw/Mn
ACE2-Fc	95.3	1.0	211.8	211.9	1.000
ACE2-Fc-TD	100.2	1.0	447.9	449.2	1.114

B. Molecular weights sedimentation co-efficient analysis

Sample	Monomeric MW (kDa)	Concentration (mg/mL)	MW (kDa)	Sed. Co (S)	f/f_0
ACE2-Fc	95.3	0.50	225	8.30	1.55
		0.25	221	8.38	1.52
		0.10	221	8.44	1.50
ACE2-Fc-TD	100.2	0.50	416	11.75	1.64
		0.25	386	11.83	1.56
		0.10	420	11.85	1.64

Note: For each sample concentration the signal-weighted sedimentation co-efficient and the estimated molecular weight of each species is shown, together with the best-fit frictional ratio for the distribution.

Supplementary Figure 2

A.

sACE2

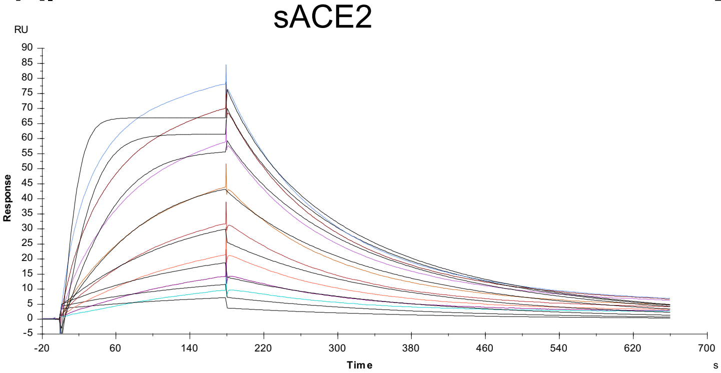

B.

ACE2-Fc

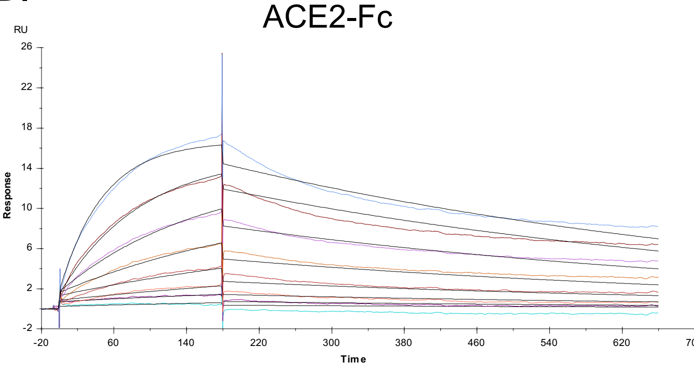

C.

ACE2-Fc-TD

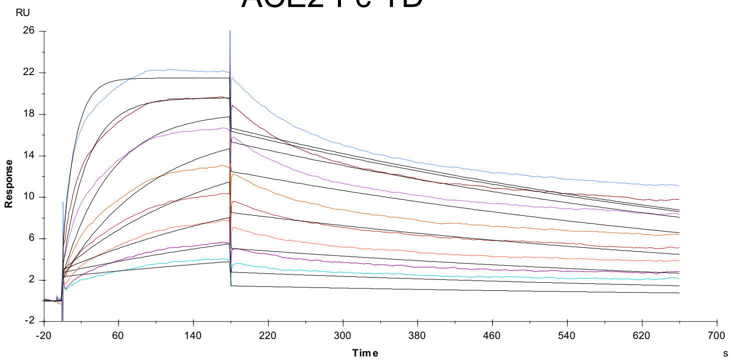

D.

	K_d (nM)	k_{on} ($M^{-1}s^{-1}$)	k_{off} (s^{-1})
**sACE2**	22 ±3	5.21×10^5 $\pm 0.49 \times 10^5$	0.012 ±0.0007
**ACE2-Fc**	21 ±7	0.92×10^5 $\pm 0.06 \times 10^5$	0.0019 ±0.0006
**ACE2-Fc-TD**	3.9 ±0.3	4.55×10^5 $\pm 1.34 \times 10^5$	0.0017 ±0.0006

Supplementary Figure S2: SPR analysis of ACE2 proteins binding to SARS-Cov-2-RBD. Sensograms for sACE2 (A), ACE2-Fc (B) and ACE2-Fc-TD (C) binding to SARS-Cov-2-RBD-Biotin are shown in colour, together with data fitting to a 1:1 kinetics model shown in black. Biotinylated SARS-Cov-2-RBD was captured on a streptavidin-coated chip and ACE2 antibodies flowed over the surface at 1.56, 3.13, 6.25, 12.5, 25, 50, 100 and 200nM. Kinetic parameters are shown in (D). Sensograms shown are representative of two independent experiments and values are the mean averages of two experiments with errors the standard deviation from the mean.

Supplementary Figure 3: sequence of the coding regions of the proteins used

Sequence shown is nucleotides and derived amino-acids of ACE2-Fc-TD. All proteins were expressed by secretion from Expi293F cells using the pTT5 vector. Leader sequence for secretion from Expi293F cells and the p53 tetramerization domain are underlined in the ACE2-Fc-TD.

ACE2-Fc-TD in pTT5

```
         10        20        30        40        50        60        70        80        90       100
 AGCTTATGGGCTGGTCCTGCATCATCCTGTTTCTGGTGGCCACAGCCACAGGCGTGCACTCTCAGAGCACCATTGAGGAACAGGCCAAGACCTTCCTGGA
 TCGAATACCCGACCAGGACGTAGTAGGACAAAGACCACCGGTGTCGGTGTCCGCACGTGAGAGTCTCGTGGTAACTCCTTGTCCGGTTCTGGAAGGACCT
      MetGlyTrpSerCysIleIleLeuPheLeuValAlaThrAlaThrGlyValHisSerGlnSerThrIleGluGluGlnAlaLysThrPheLeuAsp>

        110       120       130       140       150       160       170       180       190       200
 CAAGTTCAACCACGAGGCCGAGGACCTGTTCTACCAGTCTAGCCTGGCCAGCTGGAACTACAACACCAACATCACCGAAGAGAACGTGCAGAACATGAAC
 GTTCAAGTTGGTGCTCCGGCTCCTGGACAAGATGGTCAGATCGGACCGGTCGACCTTGATGTTGTGGTTGTAGTGGCTTCTCTTGCACGTCTTGTACTTG
  LysPheAsnHisGluAlaGluAspLeuPheTyrGlnSerSerLeuAlaSerTrpAsnTyrAsnThrAsnIleThrGluGluAsnValGlnAsnMetAsn>

        210       220       230       240       250       260       270       280       290       300
 AACGCCGGCGACAAGTGGAGCGCCTTCCTGAAAGAGCAGAGCACACTGGCCCAGATGTACCCTCTGCAAGAGATCCAGAACCTGACCGTGAAGCTCCAGC
 TTGCGGCCGCTGTTCACCTCGCGGAAGGACTTTCTCGTCTCGTGTGACCGGGTCTACATGGGAGACGTTCTCTAGGTCTTGGACTGGCACTTCGAGGTCG
 AsnAlaGlyAspLysTrpSerAlaPheLeuLysGluGlnSerThrLeuAlaGlnMetTyrProLeuGlnGluIleGlnAsnLeuThrValLysLeuGln>

        310       320       330       340       350       360       370       380       390       400
 TGCAAGCCCTCCAGCAGAATGGAAGCTCTGTGCTGAGCGAGGACAAGAGCAAGCGGCTGAACACCATCCTGAATACCATGAGCACCATCTACAGCACCGG
 ACGTTCGGGAGGTCGTCTTACCTTCGAGACACGACTCGCTCCTGTTCTCGTTCGCCGACTTGTGGTAGGACTTATGGTACTCGTGGTAGATGTCGTGGCC
LeuGlnAlaLeuGlnGlnAsnGlySerSerValLeuSerGluAspLysSerLysArgLeuAsnThrIleLeuAsnThrMetSerThrIleTyrSerThrGly>

        410       420       430       440       450       460       470       480       490       500
 CAAAGTGTGCAACCCCGACAATCCCCAAGAGTGCCTGCTGCTGGAACCCGGCCTGAATGAGATCATGGCCAACAGCCTGGACTACAACGAGAGACTGTGG
 GTTTCACACGTTGGGGCTGTTAGGGGTTCTCACGGACGACGACCTTGGGCCGGACTTACTCTAGTACCGGTTGTCGGACCTGATGTTGCTCTCTGACACC
  LysValCysAsnProAspAsnProGlnGluCysLeuLeuLeuGluProGlyLeuAsnGluIleMetAlaAsnSerLeuAspTyrAsnGluArgLeuTrp>

        510       520       530       540       550       560       570       580       590       600
 GCCTGGGAGTCTTGGAGAAGCGAAGTGGGAAAGCAGCTGCGGCCCCTGTACGAGGAATACGTGGTGCTGAAGAACGAGATGGCCAGAGCCAACCACTACG
 CGGACCCTCAGAACCTCTTCGCTTCACCCTTTCGTCGACGCCGGGGACATGCTCCTTATGCACCACGACTTCTTGCTCTACCGGTCTCGGTTGGTGATGC
 AlaTrpGluSerTrpArgSerGluValGlyLysGlnLeuArgProLeuTyrGluGluTyrValValLeuLysAsnGluMetAlaArgAlaAsnHisTyr>

        610       620       630       640       650       660       670       680       690       700
 AGGACTACGGCGACTATTGGAGAGGCGACTACGAAGTGAATGGCGTGGACGGCTACGACTACAGCAGAGGCCAGCTGATCGAGGACGTGGAACACACCTT
 TCCTGATGCCGCTGATAACCTCTCCGCTGATGCTTCACTTACCGCACCTGCCGATGCTGATGTCGTCTCCGGTCGACTAGCTCCTGCACCTTGTGTGGAA
GluAspTyrGlyAspTyrTrpArgGlyAspTyrGluValAsnGlyValAspGlyTyrAspTyrSerArgGlyGlnLeuIleGluAspValGluHisThrPhe>

        710       720       730       740       750       760       770       780       790       800
 CGAGGAAATCAAGCCTCTGTACGAGCATCTGCACGCCTACGTGCGGGCCAAGCTGATGAATGCTTACCCCAGCTACATCAGCCCCATCGGCTGTCTGCCT
 GCTCCTTTAGTTCGGAGACATGCTCGTAGACGTGCGGATGCACGCCCGGTTCGACTACTTACGAATGGGGTCGATGTAGTCGGGGTAGCCGACAGACGGA
  GluGluIleLysProLeuTyrGluHisLeuHisAlaTyrValArgAlaLysLeuMetAsnAlaTyrProSerTyrIleSerProIleGlyCysLeuPro>

        810       820       830       840       850       860       870       880       890       900
 GCTCATCTGCTGGGAGACATGTGGGGCAGATTCTGGACCAACCTGTACAGCCTGACAGTGCCCTTCGGCCAGAAACCTAACATCGACGTGACCGACGCCA
 CGAGTAGACGACCCTCTGTACACCCCGTCTAAGACCTGGTTGGACATGTCGGACTGTCACGGGAAGCCGGTCTTTGGATTGTAGCTGCACTGGCTGCGGT
 AlaHisLeuLeuGlyAspMetTrpGlyArgPheTrpThrAsnLeuTyrSerLeuThrValProPheGlyGlnLysProAsnIleAspValThrAspAla>

        910       920       930       940       950       960       970       980       990      1000
 TGGTGGATCAGGCTTGGGATGCCCAGCGGATCTTCAAAGAGGCCGAGAAGTTCTTCGTGTCCGTGGGCCTGCCTAATATGACCCAAGGCTTCTGGGAGAA
 ACCACCTAGTCCGAACCCTACGGGTCGCCTAGAAGTTTCTCCGGCTCTTCAAGAAGCACAGGCACCCGGACGGATTATACTGGGTTCCGAAGACCCTCTT
MetValAspGlnAlaTrpAspAlaGlnArgIlePheLysGluAlaGluLysPhePheValSerValGlyLeuProAsnMetThrGlnGlyPheTrpGluAsn>

       1010      1020      1030      1040      1050      1060      1070      1080      1090      1100
 CTCCATGCTGACAGACCCCGGCAATGTGCAGAAAGCCGTGTGTCATCCTACCGCCTGGGATCTCGGCAAGGGCGACTTCAGAATCCTGATGTGCACCAAA
 GAGGTACGACTGTCTGGGGCCGTTACACGTCTTTCGGCACACAGTAGGATGGCGGACCCTAGAGCCGTTCCCGCTGAAGTCTTAGGACTACACGTGGTTT
  SerMetLeuThrAspProGlyAsnValGlnLysAlaValCysHisProThrAlaTrpAspLeuGlyLysGlyAspPheArgIleLeuMetCysThrLys>

       1110      1120      1130      1140      1150      1160      1170      1180      1190      1200
 GTGACGATGGACGACTTCCTGACAGCCCACCACGAGATGGGCCACATCCAGTACGATATGGCCTACGCCGCTCAGCCCTTCCTGCTGAGAAATGGCGCCA
 CACTGCTACCTGCTGAAGGACTGTCGGGTGGTGCTCTACCCGGTGTAGGTCATGCTATACCGGATGCGGCGAGTCGGGAAGGACGACTCTTTACCGCGGT
 ValThrMetAspAspPheLeuThrAlaHisHisGluMetGlyHisIleGlnTyrAspMetAlaTyrAlaAlaGlnProPheLeuLeuArgAsnGlyAla>

       1210      1220      1230      1240      1250      1260      1270      1280      1290      1300
 ATGAGGGCTTCCACGAAGCCGTGGGAGAGATCATGAGCCTGTCTGCCGCCACACCTAAGCACCTGAAGTCTATCGGACTGCTGAGCCCCGACTTCCAAGA
 TACTCCCGAAGGTGCTTCGGCACCCTCTCTAGTACTCGGACAGACGGCGGTGTGGATTCGTGGACTTCAGATAGCCTGACGACTCGGGGCTGAAGGTTCT
AsnGluGlyPheHisGluAlaValGlyGluIleMetSerLeuSerAlaAlaThrProLysHisLeuLysSerIleGlyLeuLeuSerProAspPheGlnGlu>
```

```
      1310      1320      1330      1340      1350      1360      1370      1380      1390      1400
GGACAACGAGACAGAGATCAACTTCCTGCTCAAGCAGGCCCTGACCATCGTGGGCACACTGCCCTTTACCTACATGCTGGAAAAGTGGCGGTGGATGGTC
CCTGTTGCTCTGTCTCTAGTTGAAGGACGAGTTCGTCCGGGACTGGTAGCACCCGTGTGACGGGAAATGGATGTACGACCTTTTCACCGCCACCTACCAG
 AspAsnGluThrGluIleAsnPheLeuLeuLysGlnAlaLeuThrIleValGlyThrLeuProPheThrTyrMetLeuGluLysTrpArgTrpMetVal>

      1410      1420      1430      1440      1450      1460      1470      1480      1490      1500
TTTAAGGGCGAGATCCCCAAGGACCAGTGGATGAAGAAATGGTGGGAGATGAAGCGCGAGATCGTGGGCGTTGTGGAACCTGTGCCTCACGACGAGACAT
AAATTCCCGCTCTAGGGGTTCCTGGTCACCTACTTCTTTACCACCCTCTACTTCGCGCTCTAGCACCCGCAACACCTTGGACACGGAGTGCTGCTCTGTA
PheLysGlyGluIleProLysAspGlnTrpMetLysLysTrpTrpGluMetLysArgGluIleValGlyValValGluProValProHisAspGluThr>

      1510      1520      1530      1540      1550      1560      1570      1580      1590      1600
ACTGCGATCCTGCCAGCCTGTTTCACGTGTCCAACGACTACTCCTTCATCCGGTACTACACCCGGACACTGTACCAGTTCCAGTTTCAAGAGGCTCTGTG
TGACGCTAGGACGGTCGGACAAAGTGCACAGGTTGCTGATGAGGAAGTAGGCCATGATGTGGGCCTGTGACATGGTCAAGGTCAAAGTTCTCCGAGACAC
TyrCysAspProAlaSerLeuPheHisValSerAsnAspTyrSerPheIleArgTyrTyrThrArgThrLeuTyrGlnPheGlnPheGlnGluAlaLeuCys>

      1610      1620      1630      1640      1650      1660      1670      1680      1690      1700
CCAGGCCGCCAAGCACGAAGGACCTCTGCACAAGTGCGACATCAGCAACTCTACAGAGGCCGGACAGAAACTGTTCAACATGCTGCGGCTCGGCAAGAGC
GGTCCGGCGGTTCGTGCTTCCTGGAGACGTGTTCACGCTGTAGTCGTTGAGATGTCTCCGGCCTGTCTTTGACAAGTTGTACGACGCCGAGCCGTTCTCG
 GlnAlaAlaLysHisGluGlyProLeuHisLysCysAspIleSerAsnSerThrGluAlaGlyGlnLysLeuPheAsnMetLeuArgLeuGlyLysSer>

      1710      1720      1730      1740      1750      1760      1770      1780      1790      1800
GAGCCTTGGACACTGGCTCTGGAAAATGTCGTGGGCGCCAAGAATATGAACGTGCGGCCACTGCTGAACTACTTCGAGCCCCTGTTCACCTGGCTGAAGG
CTCGGAACCTGTGACCGAGACCTTTTACAGCACCCGCGGTTCTTATACTTGCACGCCGGTGACGACTTGATGAAGCTCGGGGACAAGTGGACCGACTTCC
GluProTrpThrLeuAlaLeuGluAsnValValGlyAlaLysAsnMetAsnValArgProLeuLeuAsnTyrPheGluProLeuPheThrTrpLeuLys>

      1810      1820      1830      1840      1850      1860      1870      1880      1890      1900
ACCAGAACAAGAACAGCTTCGTCGGCTGGTCCACCGATTGGAGCCCTTATGCTGATGAACCTAAGTCCTGCGATAAGACACATACCTGTCCCCCTTGTCC
TGGTCTTGTTCTTGTCGAAGCAGCCGACCAGGTGGCTAACCTCGGGAATACGACTACTTGGATTCAGGACGCTATTCTGTGTATGGACAGGGGGAACAGG
AspGlnAsnLysAsnSerPheValGlyTrpSerThrAspTrpSerProTyrAlaAspGluProLysSerCysAspLysThrHisThrCysProProCysPro>

      1910      1920      1930      1940      1950      1960      1970      1980      1990      2000
TGCCCCTGAACTGCTGGGCGGACCTTCCGTGTTCCTGTTCCCCCCAAAGCCCAAGGACACCCTGATGATCTCCCGGACCCCCGAAGTGACCTGCGTGGTG
ACGGGGACTTGACGACCCGCCTGGAAGGCACAAGGACAAGGGGGGTTTCGGGTTCCTGTGGGACTACTAGAGGGCCTGGGGGCTTCACTGGACGCACCAC
 AlaProGluLeuLeuGlyGlyProSerValPheLeuPheProProLysProLysAspThrLeuMetIleSerArgThrProGluValThrCysValVal>

      2010      2020      2030      2040      2050      2060      2070      2080      2090      2100
GTGGATGTGTCCCACGAGGACCCTGAAGTGAAGTTCAATTGGTACGTGGACGGCGTGGAAGTGCACAACGCCAAGACCAAGCCTAGAGAGGAACAGTACA
CACCTACACAGGGTGCTCCTGGGACTTCACTTCAAGTTAACCATGCACCTGCCGCACCTTCACGTGTTGCGGTTCTGGTTCGGATCTCTCCTTGTCATGT
ValAspValSerHisGluAspProGluValLysPheAsnTrpTyrValAspGlyValGluValHisAsnAlaLysThrLysProArgGluGluGlnTyr>

      2110      2120      2130      2140      2150      2160      2170      2180      2190      2200
ACTCCACCTACCGGGTGGTGTCCGTGCTGACCGTGCTGCACCAGGATTGGCTGAACGGCAAAGAGTACAAGTGCAAGGTGTCCAACAAGGCCCTGCCTGC
TGAGGTGGATGGCCCACCACAGGCACGACTGGCACGACGTGGTCCTAACCGACTTGCCGTTTCTCATGTTCACGTTCCACAGGTTGTTCCGGGACGGACG
AsnSerThrTyrArgValValSerValLeuThrValLeuHisGlnAspTrpLeuAsnGlyLysGluTyrLysCysLysValSerAsnLysAlaLeuProAla>

      2210      2220      2230      2240      2250      2260      2270      2280      2290      2300
CCCCATCGAAAAGACCATCTCCAAGGCCAAGGGCCAGCCCCGGGAACCCCAGGTGTACACACTGCCCCCTAGCAGGGACGAGCTGACCAAGAACCAGGTG
GGGGTAGCTTTTCTGGTAGAGGTTCCGGTTCCCGGTCGGGGCCCTTGGGGTCCACATGTGTGACGGGGGATCGTCCCTGCTCGACTGGTTCTTGGTCCAC
 ProIleGluLysThrIleSerLysAlaLysGlyGlnProArgGluProGlnValTyrThrLeuProProSerArgAspGluLeuThrLysAsnGlnVal>

      2310      2320      2330      2340      2350      2360      2370      2380      2390      2400
TCCCTGACCTGTCTCGTGAAAGGCTTCTACCCCTCCGATATCGCCGTGGAATGGGAGTCCAACGGCCAGCCTGAGAACAACTACAAGACCACCCCCCCTG
AGGGACTGGACAGAGCACTTTCCGAAGATGGGGAGGCTATAGCGGCACCTTACCCTCAGGTTGCCGGTCGGACTCTTGTTGATGTTCTGGTGGGGGGGAC
SerLeuThrCysLeuValLysGlyPheTyrProSerAspIleAlaValGluTrpGluSerAsnGlyGlnProGluAsnAsnTyrLysThrThrProPro>

      2410      2420      2430      2440      2450      2460      2470      2480      2490      2500
TGCTGGACTCCGACGGCTCATTCTTCCTGTACAGCAAGCTGACAGTGGACAAGTCCCGGTGGCAGCAGGGCAACGTGTTCTCCTGCTCCGTGATGCACGA
ACGACCTGAGGCTGCCGAGTAAGAAGGACATGTCGTTCGACTGTCACCTGTTCAGGGCCACCGTCGTCCCGTTGCACAAGAGGACGAGGCACTACGTGCT
ValLeuAspSerAspGlySerPhePheLeuTyrSerLysLeuThrValAspLysSerArgTrpGlnGlnGlyAsnValPheSerCysSerValMetHisGlu>

      2510      2520      2530      2540      2550      2560      2570      2580      2590      2600
GGCCCTGCACAACCACTACACCCAGAAGTCCCTGTCCCTGAGCCCCGGCAAGAAGAAAAAGCCCCTGGACGGCGAGTACTTCACACTGCAAATCCGGGGC
CCGGGACGTGTTGGTGATGTGGGTCTTCAGGGACAGGGACTCGGGGCCGTTCTTCTTTTTCGGGGACCTGCCGCTCATGAAGTGTGACGTTTAGGCCCCG
 AlaLeuHisAsnHisTyrThrGlnLysSerLeuSerLeuSerProGlyLysLysLysLysProLeuAspGlyGluTyrPheThrLeuGlnIleArgGly>

      2610      2620      2630      2640      2650      2660      2670      2680      2690      2700
AGAGAACGCTTCGAGATGTTCAGAGAGCTGAACGAGGCCCTGGAACTGAAGGATGCCCAGGCCGGAAAAGAGCCCGGCCACCACCATCACCACCACTGAG
TCTCTTGCGAAGCTCTACAAGTCTCTCGACTTGCTCCGGGACCTTGACTTCCTACGGGTCCGGCCTTTTCTCGGGCCGGTGGTGGTAGTGGTGGTGACTC
 ArgGluArgPheGluMetPheArgGluLeuAsnGluAlaLeuGluLeuLysAspAlaGlnAlaGlyLysGluProGlyHisHisHisHisHisHis***>
```

CR3022-Fab-TD(VH-CH1-TD + VL-CL)

CR3022 VH-CH1-TD

```
        10        20        30        40        50        60        70        80        90       100
AGCTTATGGGATGGTCTTGTATAATTCTGTTCCTGGTGGCAACAGCAACAGGAGTGCATAGCCAGATGCAGCTGGTGCAGTCTGGCACCGAAGTGAAGAA
TCGAATACCCTACCAGAACATATTAAGACAAGGACCACCGTTGTCGTTGTCCTCACGTATCGGTCTACGTCGACCACGTCAGACCGTGGCTTCACTTCTT
     MetGlyTrpSerCysIleIleLeuPheLeuValAlaThrAlaThrGlyValHisSerGlnMetGlnLeuValGlnSerGlyThrGluValLysLys>

       110       120       130       140       150       160       170       180       190       200
GCCTGGCGAGAGCCTGAAGATCAGCTGCAAAGGCAGCGGCTACGGCTTCATCACCTACTGGATCGGCTGGGTCCGACAGATGCCTGGCAAAGGCCTTGAG
CGGACCGCTCTCGGACTTCTAGTCGACGTTTCCGTCGCCGATGCCGAAGTAGTGGATGACCTAGCCGACCCAGGCTGTCTACGGACCGTTTCCGGAACTC
 ProGlyGluSerLeuLysIleSerCysLysGlySerGlyTyrGlyPheIleThrTyrTrpIleGlyTrpValArgGlnMetProGlyLysGlyLeuGlu>

       210       220       230       240       250       260       270       280       290       300
TGGATGGGCATCATCTACCCCGGCGACAGCGAGACAAGATACAGCCCTAGCTTCCAGGGCCAAGTGACCATCAGCGCCGACAAGAGCATCAACACCGCCT
ACCTACCCGTAGTAGATGGGGCCGCTGTCGCTCTGTTCTATGTCGGGATCGAAGGTCCCGGTTCACTGGTAGTCGCGGCTGTTCTCGTAGTTGTGGCGGA
TrpMetGlyIleIleTyrProGlyAspSerGluThrArgTyrSerProSerPheGlnGlyGlnValThrIleSerAlaAspLysSerIleAsnThrAla>
```

```
        310       320       330       340       350       360       370       380       390       400
ACCTTCAGTGGTCCAGCCTGAAGGCCTCTGACACCGCCATCTACTATTGTGCCGGCGGAAGCGGCATCAGCACCCCTATGGATGTTTGGGGCCAGGGCAC
TGGAAGTCACCAGGTCGGACTTCCGGAGACTGTGGCGGTAGATGATAACACGGCCGCCTTCGCCGTAGTCGTGGGGATACCTACAAACCCCGGTCCCGTG
TyrLeuGlnTrpSerSerLeuLysAlaSerAspThrAlaIleTyrTyrCysAlaGlyGlySerGlyIleSerThrProMetAspValTrpGlyGlnGlyThr>

        410       420       430       440       450       460       470       480       490       500
CACAGTGACAGTTGCCTCCACTAAGGGGCCGAGTGTTTTTCCACTTGCCCCATCCAGTAAGAGCACCTCTGGAGGAACTGCCGCCCTGGGTTGCCTTGTT
GTGTCACTGTCAACGGAGGTGATTCCCCGGCTCACAAAAAGGTGAACGGGGTAGGTCATTCTCGTGGAGACCTCCTTGACGGCGGGACCCAACGGAACAA
 ThrValThrValAlaSerThrLysGlyProSerValPheProLeuAlaProSerSerLysSerThrSerGlyGlyThrAlaAlaLeuGlyCysLeuVal>

        510       520       530       540       550       560       570       580       590       600
AAGGATTACTTCCCTGAGCCAGTAACTGTTAGCTGGAACTCTGGCGCTCTGACCAGCGGAGTGCACACCTTCCCTGCTGTGCTGCAGTCCTCAGGGCTGT
TTCCTAATGAAGGGACTCGGTCATTGACAATCGACCTTGAGACCGCGAGACTGGTCGCCTCACGTGTGGAAGGGACGACACGACGTCAGGAGTCCCGACA
LysAspTyrPheProGluProValThrValSerTrpAsnSerGlyAlaLeuThrSerGlyValHisThrPheProAlaValLeuGlnSerSerGlyLeu>

        610       620       630       640       650       660       670       680       690       700
ACTCCCTTTCTAGTGTCGTAACAGTGCCATCTTCTAGCCTGGGGACCCAGACGTACATCTGTAACGTGAATCATAAACCCAGTAACACAAAGGTAGATAA
TGAGGGAAAGATCACAGCATTGTCACGGTAGAAGATCGGACCCCTGGGTCTGCATGTAGACATTGCACTTAGTATTTGGGTCATTGTGTTTCCATCTATT
TyrSerLeuSerSerValValThrValProSerSerSerLeuGlyThrGlnThrTyrIleCysAsnValAsnHisLysProSerAsnThrLysValAspLys>

        710       720       730       740       750       760       770       780       790       800
GAAGGTTGAACCTAAGTCCTGCGATAAGACACATACCAAGAAGAAACCACTGGATGGAGAATATTTCACCCTTCAGATCCGTGGGCGTGAGCGCTTCGAG
CTTCCAACTTGGATTCAGGACGCTATTCTGTGTATGGTTCTTCTTTGGTGACCTACCTCTTATAAAGTGGGAAGTCTAGGCACCCGCACTCGCGAAGCTC
 LysValGluProLysSerCysAspLysThrHisThrLysLysLysProLeuAspGlyGluTyrPheThrLeuGlnIleArgGlyArgGluArgPheGlu>

        810       820       830       840       850       860       870       880       890       900
ATGTTCCGAGAGCTGAATGAGGCCTTGGAACTCAAGGATGCCCAGGCTGGGAAGGAGCCAGGGCACCACCACCATCACCACTGAGCGGCCGCTCGAGGCC
TACAAGGCTCTCGACTTACTCCGGAACCTTGAGTTCCTACGGGTCCGACCCTTCCTCGGTCCCGTGGTGGTGGTAGTGGTGACTCGCCGGCGAGCTCCGG
MetPheArgGluLeuAsnGluAlaLeuGluLeuLysAspAlaGlnAlaGlyLysGluProGlyHisHisHisHisHisHis***
```

CR3022 VL-CL.

```
    10        20        30        40        50        60        70        80        90        100
ATGGGATGGTCTTGTATAATTCTGTTCCTGGTGGCAACAGCAACAGGAGTGCATAGCGACATCCAGCTGACACAGAGCCCTGATAGCCTGGCCGTGTCTC
TACCCTACCAGAACATATTAAGACAAGGACCACCGTTGTCGTTGTCCTCACGTATCGCTGTAGGTCGACTGTGTCTCGGGACTATCGGACCGGCACAGAG
MetGlyTrpSerCysIleIleLeuPheLeuValAlaThrAlaThrGlyValHisSerAspIleGlnLeuThrGlnSerProAspSerLeuAlaValSer>

   110       120       130       140       150       160       170       180       190       200
TGGGAGAGAGAGCCACCATCAACTGCAAGAGCAGCCAGAGCGTGCTGTACTCCAGCATCAACAAGAACTACCTGGCCTGGTATCAGCAGAAGCCCGGCCA
ACCCTCTCTCTCGGTGGTAGTTGACGTTCTCGTCGGTCTCGCACGACATGAGGTCGTAGTTGTTCTTGATGGACCGGACCATAGTCGTCTTCGGGCCGGT
LeuGlyGluArgAlaThrIleAsnCysLysSerSerGlnSerValLeuTyrSerSerIleAsnLysAsnTyrLeuAlaTrpTyrGlnGlnLysProGlyGln>

   210       220       230       240       250       260       270       280       290       300
GCCTCCTAAGCTGCTGATCTACTGGGCCAGCACCAGAGAAAGCGGCGTGCCCGATAGATTTTCTGGCAGCGGCTCTGGCACCGACTTCACCCTGACAATT
CGGAGGATTCGACGACTAGATGACCCGGTCGTGGTCTCTTTCGCCGCACGGGCTATCTAAAAGACCGTCGCCGAGACCGTGGCTGAAGTGGGACTGTTAA
 ProProLysLeuLeuIleTyrTrpAlaSerThrArgGluSerGlyValProAspArgPheSerGlySerGlySerGlyThrAspPheThrLeuThrIle>

   310       320       330       340       350       360       370       380       390       400
AGCTCCCTGCAGGCCGAGGATGTGGCCGTGTACTACTGCCAGCAGTACTACAGCACCCCTTACACCTTTGGCCAGGGCACCAAGGTGGAAATCAAGCGTA
TCGAGGGACGTCCGGCTCCTACACCGGCACATGATGACGGTCGTCATGATGTCGTGGGGAATGTGGAAACCGGTCCCGTGGTTCCACCTTTAGTTCGCAT
SerSerLeuGlnAlaGluAspValAlaValTyrTyrCysGlnGlnTyrTyrSerThrProTyrThrPheGlyGlnGlyThrLysValGluIleLysArg>

   410       420       430       440       450       460       470       480       490       500
CGGTGGCCGCTCCCTCCGTGTTCATCTTCCCACCTTCCGACGAGCAGCTGAAGTCCGGCACCGCTTCTGTCGTGTGCCTGCTGAACAACTTCTACCCCCG
GCCACCGGCGAGGGAGGCACAAGTAGAAGGGTGGAAGGCTGCTCGTCGACTTCAGGCCGTGGCGAAGACAGCACACGGACGACTTGTTGAAGATGGGGGC
ThrValAlaAlaProSerValPheIlePheProProSerAspGluGlnLeuLysSerGlyThrAlaSerValValCysLeuLeuAsnAsnPheTyrProArg>

   510       520       530       540       550       560       570       580       590       600
CGAGGCCAAGGTGCAGTGGAAGGTGGACAACGCCCTGCAGTCCGGCAACTCCCAGGAATCCGTGACCGAGCAGGACTCCAAGGACAGCACCTACTCCCTG
GCTCCGGTTCCACGTCACCTTCCACCTGTTGCGGGACGTCAGGCCGTTGAGGGTCCTTAGGCACTGGCTCGTCCTGAGGTTCCTGTCGTGGATGAGGGAC
 GluAlaLysValGlnTrpLysValAspAsnAlaLeuGlnSerGlyAsnSerGlnGluSerValThrGluGlnAspSerLysAspSerThrTyrSerLeu>

   610       620       630       640       650       660       670       680       690       700
TCCTCCACCCTGACCCTGTCCAAGGCCGACTACGAGAAGCACAAGGTGTACGCCTGCGAAGTGACCCACCAGGGCCTGTCTAGCCCCGTGACCAAGTCTT
AGGAGGTGGGACTGGGACAGGTTCCGGCTGATGCTCTTCGTGTTCCACATGCGGACGCTTCACTGGGTGGTCCCGGACAGATCGGGGCACTGGTTCAGAA
SerSerThrLeuThrLeuSerLysAlaAspTyrGluLysHisLysValTyrAlaCysGluValThrHisGlnGlyLeuSerSerProValThrLysSer>

   710       720
TCAACCGGGGCGAGTGT
AGTTGGCCCCGCTCACA
PheAsnArgGlyGluCys>
```

CR3022-scFv-TD

```
    10        20        30        40        50        60        70        80        90        100
ATGGGATGGTCTTGTATAATTCTGTTCCTGGTGGCAACAGCAACAGGAGTGCATAGCCAGATGCAGCTGGTGCAGTCTGGCACCGAAGTGAAGAAGCCTG
TACCCTACCAGAACATATTAAGACAAGGACCACCGTTGTCGTTGTCCTCACGTATCGGTCTACGTCGACCACGTCAGACCGTGGCTTCACTTCTTCGGAC
MetGlyTrpSerCysIleIleLeuPheLeuValAlaThrAlaThrGlyValHisSerGlnMetGlnLeuValGlnSerGlyThrGluValLysLysPro>

   110       120       130       140       150       160       170       180       190       200
GCGAGAGCCTGAAGATCAGCTGCAAAGGCAGCGGCTACGGCTTCATCACCTACTGGATCGGCTGGGTCCGACAGATGCCTGGCAAAGGCCTTGAGTGGAT
CGCTCTCGGACTTCTAGTCGACGTTTCCGTCGCCGATGCCGAAGTAGTGGATGACCTAGCCGACCCAGGCTGTCTACGGACCGTTTCCGGAACTCACCTA
GlyGluSerLeuLysIleSerCysLysGlySerGlyTyrGlyPheIleThrTyrTrpIleGlyTrpValArgGlnMetProGlyLysGlyLeuGluTrpMet>
```

```
  210        220        230        240        250        260        270        280        290        300
GGGCATCATCTACCCCGGCGACAGCGAGACAAGATACAGCCCTAGCTTCCAGGGCCAAGTGACCATCAGCGCCGACAAGAGCATCAACACCGCCTACCTT
CCCGTAGTAGATGGGGCCGCTGTCGCTCTGTTCTATGTCGGGATCGAAGGTCCCGGTTCACTGGTAGTCGCGGCTGTTCTCGTAGTTGTGGCGGATGGAA
 GlyIleIleTyrProGlyAspSerGluThrArgTyrSerProSerPheGlnGlyGlnValThrIleSerAlaAspLysSerIleAsnThrAlaTyrLeu>

  310        320        330        340        350        360        370        380        390        400
CAGTGGTCCAGCCTGAAGGCCTCTGACACCGCCATCTACTATTGTGCCGGCGGAAGCGGCATCAGCACCCCTATGGATGTTTGGGGCCAGGGCACCACAG
GTCACCAGGTCGGACTTCCGGAGACTGTGGCGGTAGATGATAACACGGCCGCCTTCGCCGTAGTCGTGGGGATACCTACAAACCCCGGTCCCGTGGTGTC
GlnTrpSerSerLeuLysAlaSerAspThrAlaIleTyrTyrCysAlaGlyGlySerGlyIleSerThrProMetAspValTrpGlyGlnGlyThrThr>

  410        420        430        440        450        460        470        480        490        500
TGACAGTTGGCGGAGGTGGAAGCGGAGGCGGAGGATCTGGTGGTGGTGGATCTGACATCCAGCTGACACAGAGCCCTGATAGCCTGGCCGTGTCTCTGGG
ACTGTCAACCGCCTCCACCTTCGCCTCCGCCTCCTAGACCACCACCACCTAGACTGTAGGTCGACTGTGTCTCGGGACTATCGGACCGGCACAGAGACCC
ValThrValGlyGlyGlyGlySerGlyGlyGlyGlySerGlyGlyGlyGlySerAspIleGlnLeuThrGlnSerProAspSerLeuAlaValSerLeuGly>
_______________________________________________320 aa_________________________________________________>

  510        520        530        540        550        560        570        580        590        600
AGAGAGAGCCACCATCAACTGCAAGAGCAGCCAGAGCGTGCTGTACTCCAGCATCAACAAGAACTACCTGGCCTGGTATCAGCAGAAGCCCGGCCAGCCT
TCTCTCTCGGTGGTAGTTGACGTTCTCGTCGGTCTCGCACGACATGAGGTCGTAGTTGTTCTTGATGGACCGGACCATAGTCGTCTTCGGGCCGGTCGGA
 GluArgAlaThrIleAsnCysLysSerSerGlnSerValLeuTyrSerSerIleAsnLysAsnTyrLeuAlaTrpTyrGlnGlnLysProGlyGlnPro>

  610        620        630        640        650        660        670        680        690        700
CCTAAGCTGCTGATCTACTGGGCCAGCACCAGAGAAAGCGGCGTGCCCGATAGATTTTCTGGCAGCGGCTCTGGCACCGACTTCACCCTGACAATTAGCT
GGATTCGACGACTAGATGACCCGGTCGTGGTCTCTTTCGCCGCACGGGCTATCTAAAAGACCGTCGCCGAGACCGTGGCTGAAGTGGGACTGTTAATCGA
ProLysLeuLeuIleTyrTrpAlaSerThrArgGluSerGlyValProAspArgPheSerGlySerGlySerGlyThrAspPheThrLeuThrIleSer>

  710        720        730        740        750        760        770        780        790        800
CCCTGCAGGCCGAGGATGTGGCCGTGTACTACTGCCAGCAGTACTACAGCACCCCTTACACCTTTGGCCAGGGCACCAAGGTGGAAATCAAGCGTAAGAA
GGGACGTCCGGCTCCTACACCGGCACATGATGACGGTCGTCATGATGTCGTGGGGAATGTGGAAACCGGTCCCGTGGTTCCACCTTTAGTTCGCATTCTT
SerLeuGlnAlaGluAspValAlaValTyrTyrCysGlnGlnTyrTyrSerThrProTyrThrPheGlyGlnGlyThrLysValGluIleLysArgLysLys>

  810        820        830        840        850        860        870        880        890        900
GAAACCACTGGATGGAGAATATTTCACCCTTCAGATCCGTGGGCGTGAGCGCTTCGAGATGTTCCGAGAGCTGAATGAGGCCTTGGAACTCAAGGATGCC
CTTTGGTGACCTACCTCTTATAAAGTGGGAAGTCTAGGCACCCGCACTCGCGAAGCTCTACAAGGCTCTCGACTTACTCCGGAACCTTGAGTTCCTACGG
 LysProLeuAspGlyGluTyrPheThrLeuGlnIleArgGlyArgGluArgPheGluMetPheArgGluLeuAsnGluAlaLeuGluLeuLysAspAla>

  910        920        930        940        950        960
CAGGCTGGGAAGGAGCCAGGGGACTACAAGGACGACGACGACAAACACCACCATCACCACCAC
GTCCGACCCTTCCTCGGTCCCCTGATGTTCCTGCTGCTGCTGTTTGTGGTGGTAGTGGTGGTG
GlnAlaGlyLysGluProGlyAspTyrLysAspAspAspAspLysHisHisHisHisHisHis>
                          Flag Tag
```

CR3014-Fab-TD (VH-CH1-TD + VL-CL)

CR3014 VH1-CH1-TD

```
   10         20         30         40         50         60         70         80         90        100
ATGGGATGGTCTTGTATAATTCTGTTCCTGGTGGCAACAGCAACAGGAGTGCATAGCGAGGTGCAGCTGGTTGAATCTGGCGGAGGACTGGTTCAGCCTG
TACCCTACCAGAACATATTAAGACAAGGACCACCGTTGTCGTTGTCCTCACGTATCGCTCCACGTCGACCAACTTAGACCGCCTCCTGACCAAGTCGGAC
MetGlyTrpSerCysIleIleLeuPheLeuValAlaThrAlaThrGlyValHisSerGluValGlnLeuValGluSerGlyGlyGlyLeuValGlnPro>
  110        120        130        140        150        160        170        180        190        200
GCGGATCTCTGAGACTGTCTTGTGCCGCCAGCGGCTTCACCTTCAGCGACCACTATATGGACTGGGTCCGACAGGCCCCTGGCAAAGGACTTGAGTGGGT
CGCCTAGAGACTCTGACAGAACACGGCGGTCGCCGAAGTGGAAGTCGCTGGTGATATACCTGACCCAGGCTGTCCGGGGACCGTTTCCTGAACTCACCCA
GlyGlySerLeuArgLeuSerCysAlaAlaSerGlyPheThrPheSerAspHisTyrMetAspTrpValArgGlnAlaProGlyLysGlyLeuGluTrpVal>

  210        220        230        240        250        260        270        280        290        300
CGGACGGACCAGAAACAAGGCCAACAGCTACACCACAGAGTACGCCGCCTCTGTGAAGGGCAGATTCACCATCAGCCGGGACGACAGCAAGAACAGCCTG
GCCTGCCTGGTCTTTGTTCCGGTTGTCGATGTGGTGTCTCATGCGGCGGAGACACTTCCCGTCTAAGTGGTAGTCGGCCCTGCTGTCGTTCTTGTCGGAC
 GlyArgThrArgAsnLysAlaAsnSerTyrThrThrGluTyrAlaAlaSerValLysGlyArgPheThrIleSerArgAspAspSerLysAsnSerLeu>

  310        320        330        340        350        360        370        380        390        400
TACCTTCAGATGAACTCCCTGAAAACCGAGGACACCGCCGTGTACTACTGCGCCAGAGGCATCAGCCCCTTCTACTTCGATTATTGGGGCCAGGGCACCC
ATGGAAGTCTACTTGAGGGACTTTTGGCTCCTGTGGCGGCACATGATGACGCGGTCTCCGTAGTCGGGGAAGATGAAGCTAATAACCCCGGTCCCGTGGG
TyrLeuGlnMetAsnSerLeuLysThrGluAspThrAlaValTyrTyrCysAlaArgGlyIleSerProPheTyrPheAspTyrTrpGlyGlnGlyThr>

  410        420        430        440        450        460        470        480        490        500
TGGTCACAGTTGCCTCCACTAAGGGGCCGAGTGTTTTTCCACTTGCCCCATCCAGTAAGAGCACCTCTGGAGGAACTGCCGCCCTGGGTTGCCTTGTTAA
ACCAGTGTCAACGGAGGTGATTCCCCGGCTCACAAAAAGGTGAACGGGGTAGGTCATTCTCGTGGAGACCTCCTTGACGGCGGGACCCAACGGAACAATT
LeuValThrValAlaSerThrLysGlyProSerValPheProLeuAlaProSerSerLysSerThrSerGlyGlyThrAlaAlaLeuGlyCysLeuValLys>

  510        520        530        540        550        560        570        580        590        600
GGATTACTTCCCTGAGCCAGTAACTGTTAGCTGGAACTCTGGCGCTCTGACCAGCGGAGTGCACACCTTCCCTGCTGTGCTGCAGTCCTCAGGGCTGTAC
CCTAATGAAGGGACTCGGTCATTGACAATCGACCTTGAGACCGCGAGACTGGTCGCCTCACGTGTGGAAGGGACGACACGACGTCAGGAGTCCCGACATG
 AspTyrPheProGluProValThrValSerTrpAsnSerGlyAlaLeuThrSerGlyValHisThrPheProAlaValLeuGlnSerSerGlyLeuTyr>

  610        620        630        640        650        660        670        680        690        700
TCCCTTTCTAGTGTCGTAACAGTGCCATCTTCTAGCCTGGGGACCCAGACGTACATCTGTAACGTGAATCATAAACCCAGTAACACAAAGGTAGATAAGA
AGGGAAAGATCACAGCATTGTCACGGTAGAAGATCGGACCCCTGGGTCTGCATGTAGACATTGCACTTAGTATTTGGGTCATTGTGTTTCCATCTATTCT
SerLeuSerSerValValThrValProSerSerSerLeuGlyThrGlnThrTyrIleCysAsnValAsnHisLysProSerAsnThrLysValAspLys>

  710        720        730        740        750        760        770        780        790        800
AGGTTGAACCTAAGTCCTGCGATAAGACACATACCAAGAAGAAACCACTGGATGGAGAATATTTCACCCTTCAGATCCGTGGGCGTGAGCGCTTCGAGAT
TCCAACTTGGATTCAGGACGCTATTCTGTGTATGGTTCTTCTTTGGTGACCTACCTCTTATAAAGTGGGAAGTCTAGGCACCCGCACTCGCGAAGCTCTA
LysValGluProLysSerCysAspLysThrHisThrLysLysLysProLeuAspGlyGluTyrPheThrLeuGlnIleArgGlyArgGluArgPheGluMet>
```

```
   810        820        830        840        850        860        870        880
 GTTCCGAGAGCTGAATGAGGCCTTGGAACTCAAGGATGCCCAGGCTGGGAAGGAGCCAGGGCACCACCACCATCACCAC
 CAAGGCTCTCGACTTACTCCGGAACCTTGAGTTCCTACGGGTCCGACCCTTCCTCGGTCCCGTGGTGGTGGTAGTGGTG
  PheArgGluLeuAsnGluAlaLeuGluLeuLysAspAlaGlnAlaGlyLysGluProGlyHisHisHisHisHisHis>
```

CR3014 VL-CL

```
    10         20         30         40         50         60         70         80         90        100
 ATGGGATGGTCTTGTATAATTCTGTTCCTGGTGGCAACAGCAACAGGAGTGCATAGCGAGCTGACACAGAGCCCTTCTAGCCTGTCTGCCAGCGTGGGCG
 TACCCTACCAGAACATATTAAGACAAGGACCACCGTTGTCGTTGTCCTCACGTATCGCTCGACTGTGTCTCGGGAAGATCGGACAGACGGTCGCACCCGC
 MetGlyTrpSerCysIleIleLeuPheLeuValAlaThrAlaThrGlyValHisSerGluLeuThrGlnSerProSerSerLeuSerAlaSerValGly>

   110        120        130        140        150        160        170        180        190        200
 ACAGAGTGACCATCACATGTAGAGCCAGCCAGAGCATCAGCAGCTACCTGAACTGGTATCAGCAGAAGCCCGGCAAGGCCCCTAAACTGCTGATCTATGC
 TGTCTCACTGGTAGTGTACATCTCGGTCGGTCTCGTAGTCGTCGATGGACTTGACCATAGTCGTCTTCGGGCCGTTCCGGGGATTTGACGACTAGATACG
AspArgValThrIleThrCysArgAlaSerGlnSerIleSerSerTyrLeuAsnTrpTyrGlnGlnLysProGlyLysAlaProLysLeuLeuIleTyrAla>

   210        220        230        240        250        260        270        280        290        300
 CGCCAGCTCTCTGCAGTCTGGCGTGCCATCTAGATTTTCCGGCAGCGGCTCTGGCACCGACTTCACCCTGACCATATCTAGCCTGCAGCCTGAGGACTTC
 GCGGTCGAGAGACGTCAGACCGCACGGTAGATCTAAAAGGCCGTCGCCGAGACCGTGGCTGAAGTGGGACTGGTATAGATCGGACGTCGGACTCCTGAAG
  AlaSerSerLeuGlnSerGlyValProSerArgPheSerGlySerGlySerGlyThrAspPheThrLeuThrIleSerSerLeuGlnProGluAspPhe>

   310        320        330        340        350        360        370        380        390        400
 GCCACCTACTACTGCCAGCAGAGCTACAGCACCCCTCCTACATTTGGCCAGGGCACCAAGGTGGAAATCAAGCGTACGGTGGCCGCTCCCTCCGTGTTCA
 CGGTGGATGATGACGGTCGTCTCGATGTCGTGGGGAGGATGTAAACCGGTCCCGTGGTTCCACCTTTAGTTCGCATGCCACCGGCGAGGGAGGCACAAGT
 AlaThrTyrTyrCysGlnGlnSerTyrSerThrProProThrPheGlyGlnGlyThrLysValGluIleLysArgThrValAlaAlaProSerValPhe>

   410        420        430        440        450        460        470        480        490        500
 TCTTCCCACCTTCCGACGAGCAGCTGAAGTCCGGCACCGCTTCTGTCGTGTGCCTGCTGAACAACTTCTACCCCCGCGAGGCCAAGGTGCAGTGGAAGGT
 AGAAGGGTGGAAGGCTGCTCGTCGACTTCAGGCCGTGGCGAAGACAGCACACGGACGACTTGTTGAAGATGGGGGCGCTCCGGTTCCACGTCACCTTCCA
IlePheProProSerAspGluGlnLeuLysSerGlyThrAlaSerValValCysLeuLeuAsnAsnPheTyrProArgGluAlaLysValGlnTrpLysVal>

   510        520        530        540        550        560        570        580        590        600
 GGACAACGCCCTGCAGTCCGGCAACTCCCAGGAATCCGTGACCGAGCAGGACTCCAAGGACAGCACCTACTCCCTGTCCTCCACCCTGACCCTGTCCAAG
 CCTGTTGCGGGACGTCAGGCCGTTGAGGGTCCTTAGGCACTGGCTCGTCCTGAGGTTCCTGTCGTGGATGAGGGACAGGAGGTGGGACTGGGACAGGTTC
  AspAsnAlaLeuGlnSerGlyAsnSerGlnGluSerValThrGluGlnAspSerLysAspSerThrTyrSerLeuSerSerThrLeuThrLeuSerLys>

   610        620        630        640        650        660        670        680        690
 GCCGACTACGAGAAGCACAAGGTGTACGCCTGCGAAGTGACCCACCAGGGCCTGTCTAGCCCCGTGACCAAGTCTTTCAACCGGGGCGAGTGT
 CGGCTGATGCTCTTCGTGTTCCACATGCGGACGCTTCACTGGGTGGTCCCGGACAGATCGGGGCACTGGTTCAGAAAGTTGGCCCCGCTCACA
 AlaAspTyrGluLysHisLysValTyrAlaCysGluValThrHisGlnGlyLeuSerSerProValThrLysSerPheAsnArgGlyGluCys>
```

H4-Fab-TD (VH-CH-TD + VL-CL)

H4 VH1-CH1-TD

```
    10         20         30         40         50         60         70         80         90        100
 ATGGGCTGGTCCTGCATCATCCTGTTTCTGGTGGCCACAGCCACAGGCGTGCACTCTCAGGTTCAGCTGGTTCAGTCTGGCGCCGAAGTGAAGAAACCTG
 TACCCGACCAGGACGTAGTAGGACAAAGACCACCGGTGTCGGTGTCCGCACGTGAGAGTCCAAGTCGACCAAGTCAGACCGCGGCTTCACTTCTTTGGAC
 MetGlyTrpSerCysIleIleLeuPheLeuValAlaThrAlaThrGlyValHisSerGlnValGlnLeuValGlnSerGlyAlaGluValLysLysPro>

   110        120        130        140        150        160        170        180        190        200
 GCGCCTCTGTGAAGGTGTCCTGCAAGGCCAGCGGCTACACCTTTACCGGCTACTACATGCACTGGGTCCGACAGGCTCCAGGACAGGGACTTGAGTGGAT
 CGCGGAGACACTTCCACAGGACGTTCCGGTCGCCGATGTGGAAATGGCCGATGATGTACGTGACCCAGGCTGTCCGAGGTCCTGTCCCTGAACTCACCTA
GlyAlaSerValLysValSerCysLysAlaSerGlyTyrThrPheThrGlyTyrTyrMetHisTrpValArgGlnAlaProGlyGlnGlyLeuGluTrpMet>

   210        220        230        240        250        260        270        280        290        300
 GGGCAGAATCAACCCCAATAGCGGCGGCACCAACTACGCCCAGAAATTCCAGGGCAGAGTGACCATGACCAGAGACACCAGCATCAGCACCGCCTACATG
 CCCGTCTTAGTTGGGGTTATCGCCGCCGTGGTTGATGCGGGTCTTTAAGGTCCCGTCTCACTGGTACTGGTCTCTGTGGTCGTAGTCGTGGCGGATGTAC
  GlyArgIleAsnProAsnSerGlyGlyThrAsnTyrAlaGlnLysPheGlnGlyArgValThrMetThrArgAspThrSerIleSerThrAlaTyrMet>

   310        320        330        340        350        360        370        380        390        400
 GAACTGAGCCGGCTGAGATCCGATGACACCGCCGTGTACTACTGCGCCAGAGTGCCTTACTGTAGCAGCACCAGCTGCCACAGAGACTGGTACTTCGACC
 CTTGACTCGGCCGACTCTAGGCTACTGTGGCGGCACATGATGACGCGGTCTCACGGAATGACATCGTCGTGGTCGACGGTGTCTCTGACCATGAAGCTGG
 GluLeuSerArgLeuArgSerAspAspThrAlaValTyrTyrCysAlaArgValProTyrCysSerSerThrSerCysHisArgAspTrpTyrPheAsp>

   410        420        430        440        450        460        470        480        490        500
 TGTGGGGCAGAGGCACACTGGTCACAGTGTCTAGCGCCAGCACAAAGGGCCCTAGCGTTTTCCCACTGGCTCCCAGCAGCAAGTCTACCTCTGGTGGAAC
 ACACCCCGTCTCCGTGTGACCAGTGTCACAGATCGCGGTCGTGTTTCCCGGGATCGCAAAAGGGTGACCGAGGGTCGTCGTTCAGATGGAGACCACCTTG
LeuTrpGlyArgGlyThrLeuValThrValSerSerAlaSerThrLysGlyProSerValPheProLeuAlaProSerSerLysSerThrSerGlyGlyThr>

   510        520        530        540        550        560        570        580        590        600
 AGCCGCTCTGGGCTGCCTGGTCAAGGATTACTTTCCCGAGCCTGTGACCGTGTCCTGGAATAGCGGAGCACTGACAAGCGGCGTGCACACCTTTCCAGCT
 TCGGCGAGACCCGACGGACCAGTTCCTAATGAAAGGGCTCGGACACTGGCACAGGACCTTATCGCCTCGTGACTGTTCGCCGCACGTGTGGAAAGGTCGA
  AlaAlaLeuGlyCysLeuValLysAspTyrPheProGluProValThrValSerTrpAsnSerGlyAlaLeuThrSerGlyValHisThrPheProAla>

   610        620        630        640        650        660        670        680        690        700
 GTGCTGCAAAGCAGCGGCCTGTACTCTCTGAGCAGCGTCGTGACAGTGCCTAGCAGCTCTCTGGGCACCCAGACCTACATCTGCAATGTGAACCACAAGC
 CACGACGTTTCGTCGCCGGACATGAGAGACTCGTCGCAGCACTGTCACGGATCGTCGAGAGACCCGTGGGTCTGGATGTAGACGTTACACTTGGTGTTCG
 ValLeuGlnSerSerGlyLeuTyrSerLeuSerSerValValThrValProSerSerSerLeuGlyThrGlnThrTyrIleCysAsnValAsnHisLys>

   710        720        730        740        750        760        770        780        790        800
 CTAGCAACACCAAGGTGGACAAGAAGGTGGAACCCAAGAGCTGCGACAAGACCCACACCAAGAAGAAGCCTCTGGACGGCGAGTACTTCACTCTGCAGAT
 GATCGTTGTGGTTCCACCTGTTCTTCCACCTTGGGTTCTCGACGCTGTTCTGGGTGTGGTTCTTCTTCGGAGACCTGCCGCTCATGAAGTGAGACGTCTA
ProSerAsnThrLysValAspLysLysValGluProLysSerCysAspLysThrHisThrLysLysLysProLeuAspGlyGluTyrPheThrLeuGlnIle>
```

```
   810       820       830       840       850       860       870       880       890       900
 CCGGGGCAGAGAACGCTTCGAGATGTTTAGAGAGCTGAACGAGGCCCTGGAACTGAAGGATGCCCAGGCCGGAAAAGAGCCTGGACACCACCATCACCAT
 GGCCCCGTCTCTTGCGAAGCTCTACAAATCTCTCGACTTGCTCCGGGACCTTGACTTCCTACGGGTCCGGCCTTTTCTCGGACCTGTGGTGGTAGTGGTA
  ArgGlyArgGluArgPheGluMetPheArgGluLeuAsnGluAlaLeuGluLeuLysAspAlaGlnAlaGlyLysGluProGly HisHisHisHisHis>

 CAC
 GTG
 His>
 ___>
```

H4 VL-CL

```
    10        20        30        40        50        60        70        80        90       100
 ATGGGCTGGTCCTGCATCATCCTGTTTCTGGTGGCCACAGCCACAGGCGTGCACAGCGATATCCAGATGACACAGAGCCCTCTGAGCCTGCCTGTGACAC
 TACCCGACCAGGACGTAGTAGGACAAAGACCACCGGTGTCGGTGTCCGCACGTGTCGCTATAGGTCTACTGTGTCTCGGGAGACTCGGACGGACACTGTG
 MetGlyTrpSerCysIleIleLeuPheLeuValAlaThrAlaThrGlyValHisSerAspIleGlnMetThrGlnSerProLeuSerLeuProValThr>

   110       120       130       140       150       160       170       180       190       200
 CTGGCGAACCTGCCAGCATCAGCTGTAGAAGCAGCCAGAGCCTGCTGGACAGCGACGACGGCAATACCTACCTGGACTGGTATCTGCAGAAGCCCGGCCA
 GACCGCTTGGACGGTCGTAGTCGACATCTTCGTCGGTCTCGGACGACCTGTCGCTGCTGCCGTTATGGATGGACCTGACCATAGACGTCTTCGGGCCGGT
ProGlyGluProAlaSerIleSerCysArgSerSerGlnSerLeuLeuAspSerAspAspGlyAsnThrTyrLeuAspTrpTyrLeuGlnLysProGlyGln>

   210       220       230       240       250       260       270       280       290       300
 GTCTCCTCAGCTGCTGATCTACACCCTGAGCTACAGAGCCAGCGGCGTGCCCGATAGATTTTCTGGCTCTGGCAGCGGCACCGACTTCACCCTGAAGATC
 CAGAGGAGTCGACGACTAGATGTGGGACTCGATGTCTCGGTCGCCGCACGGGCTATCTAAAAGACCGAGACCGTCGCCGTGGCTGAAGTGGGACTTCTAG
  SerProGlnLeuLeuIleTyrThrLeuSerTyrArgAlaSerGlyValProAspArgPheSerGlySerGlySerGlyThrAspPheThrLeuLysIle>

   310       320       330       340       350       360       370       380       390       400
 TCTAGAGTGGAAGCCGAGGACGTGGGCGTGTACTACTGTATGCAGCGGATCGAGTTCCCTCTGACCTTTGGCGGCGGAACAAAGGTGGAAATCAAGCGGA
 AGATCTCACCTTCGGCTCCTGCACCCGCACATGATGACATACGTCGCCTAGCTCAAGGGAGACTGGAAACCGCCGCCTTGTTTCCACCTTTAGTTCGCCT
 SerArgValGluAlaGluAspValGlyValTyrTyrCysMetGlnArgIleGluPheProLeuThrPheGlyGlyGlyThrLysValGluIleLysArg>

   410       420       430       440       450       460       470       480       490       500
 CAGTGGCCGCTCCTAGCGTGTTCATCTTTCCACCTAGCGACGAGCAGCTGAAGTCTGGCACAGCCTCTGTCGTGTGCCTGCTGAACAACTTCTACCCCAG
 GTCACCGGCGAGGATCGCACAAGTAGAAAGGTGGATCGCTGCTCGTCGACTTCAGACCGTGTCGGAGACAGCACACGGACGACTTGTTGAAGATGGGGTC
ThrValAlaAlaProSerValPheIlePheProProSerAspGluGlnLeuLysSerGlyThrAlaSerValValCysLeuLeuAsnAsnPheTyrProArg>
   510       520       530       540       550       560       570       580       590       600
 AGAAGCCAAGGTGCAGTGGAAGGTGGACAACGCCCTGCAGAGCGGCAATAGCCAAGAGAGCGTGACCGAGCAGGACAGCAAGGACTCTACCTACAGCCTG
 TCTTCGGTTCCACGTCACCTTCCACCTGTTGCGGGACGTCTCGCCGTTATCGGTTCTCTCGCACTGGCTCGTCCTGTCGTTCCTGAGATGGATGTCGGAC
  GluAlaLysValGlnTrpLysValAspAsnAlaLeuGlnSerGlyAsnSerGlnGluSerValThrGluGlnAspSerLysAspSerThrTyrSerLeu>
   610       620       630       640       650       660       670       680       690       700
 AGCAGCACCCTGACACTGAGCAAGGCCGACTACGAGAAGCACAAAGTGTACGCCTGCGAAGTGACCCACCAGGGCCTTTCTAGCCCTGTGACCAAGAGCT
 TCGTCGTGGGACTGTGACTCGTTCCGGCTGATGCTCTTCGTGTTTCACATGCGGACGCTTCACTGGGTGGTCCCGGAAAGATCGGGACACTGGTTCTCGA
 SerSerThrLeuThrLeuSerLysAlaAspTyrGluLysHisLysValTyrAlaCysGluValThrHisGlnGlyLeuSerSerProValThrLysSer>
   710       720
 TCAACCGGGGCGAATGTT
 AGTTGGCCCCGCTTACAA
PheAsnArgGlyGluCys>
```

H4-scFv-TD

```
    10        20        30        40        50        60        70        80        90       100
 ATGGGCTGGTCCTGCATCATCCTGTTTCTGGTGGCCACAGCCACAGGCGTGCACTCTCAGGTTCAGCTGGTTCAGTCTGGCGCCGAAGTGAAGAAACCTG
 TACCCGACCAGGACGTAGTAGGACAAAGACCACCGGTGTCGGTGTCCGCACGTGAGAGTCCAAGTCGACCAAGTCAGACCGCGGCTTCACTTCTTTGGAC
 MetGlyTrpSerCysIleIleLeuPheLeuValAlaThrAlaThrGlyValHisSerGlnValGlnLeuValGlnSerGlyAlaGluValLysLysPro>

   110       120       130       140       150       160       170       180       190       200
 GCGCCTCTGTGAAGGTGTCCTGCAAGGCCAGCGGCTACACCTTTACCGGCTACTACATGCACTGGGTCCGACAGGCTCCAGGACAGGGACTTGAGTGGAT
 CGCGGAGACACTTCCACAGGACGTTCCGGTCGCCGATGTGGAAATGGCCGATGATGTACGTGACCCAGGCTGTCCGAGGTCCTGTCCCTGAACTCACCTA
GlyAlaSerValLysValSerCysLysAlaSerGlyTyrThrPheThrGlyTyrTyrMetHisTrpValArgGlnAlaProGlyGlnGlyLeuGluTrpMet>

   210       220       230       240       250       260       270       280       290       300
 GGGCAGAATCAACCCCAATAGCGGCGGCACCAACTACGCCCAGAAATTCCAGGGCAGAGTGACCATGACCAGAGACACCAGCATCAGCACCGCCTACATG
 CCCGTCTTAGTTGGGGTTATCGCCGCCGTGGTTGATGCGGGTCTTTAAGGTCCCGTCTCACTGGTACTGGTCTCTGTGGTCGTAGTCGTGGCGGATGTAC
  GlyArgIleAsnProAsnSerGlyGlyThrAsnTyrAlaGlnLysPheGlnGlyArgValThrMetThrArgAspThrSerIleSerThrAlaTyrMet>

   310       320       330       340       350       360       370       380       390       400
 GAACTGAGCCGGCTGAGATCCGATGACACCGCCGTGTACTACTGCGCCAGAGTGCCTTACTGTAGCAGCACCAGCTGCCACAGAGACTGGTACTTCGACC
 CTTGACTCGGCCGACTCTAGGCTACTGTGGCGGCACATGATGACGCGGTCTCACGGAATGACATCGTCGTGGTCGACGGTGTCTCTGACCATGAAGCTGG
 GluLeuSerArgLeuArgSerAspAspThrAlaValTyrTyrCysAlaArgValProTyrCysSerSerThrSerCysHisArgAspTrpTyrPheAsp>

   410       420       430       440       450       460       470       480       490       500
 TGTGGGGCAGAGGCACACTGGTCACAGTTTCTAGCGGAGGCGGAGGATCTGGTGGCGGAGGAAGTGGCGGAGGCGGTTCTGATATCCAGATGACACAGAG
 ACACCCCGTCTCCGTGTGACCAGTGTCAAAGATCGCCTCCGCCTCCTAGACCACCGCCTCCTTCACCGCCTCCGCCAAGACTATAGGTCTACTGTGTCTC
LeuTrpGlyArgGlyThrLeuValThrValSerSerGlyGlyGlyGlySerGlyGlyGlyGlySerGlyGlyGlyGlySerAspIleGlnMetThrGlnSer>

   510       520       530       540       550       560       570       580       590       600
 CCCTCTGAGCCTGCCTGTGACACCTGGCGAACCTGCCAGCATCTCCTGTAGAAGCTCTCAGAGCCTGCTGGACAGCGACGACGGCAACACATACCTGGAC
 GGGAGACTCGGACGGACACTGTGGACCGCTTGGACGGTCGTAGAGGACATCTTCGAGAGTCTCGGACGACCTGTCGCTGCTGCCGTTGTGTATGGACCTG
  ProLeuSerLeuProValThrProGlyGluProAlaSerIleSerCysArgSerSerGlnSerLeuLeuAspSerAspAspGlyAsnThrTyrLeuAsp>

   610       620       630       640       650       660       670       680       690       700
 TGGTATCTGCAGAAGCCCGGCCAGTCTCCTCAGCTGCTGATCTACACCCTGAGCTACAGAGCCAGCGGCGTGCCCGATAGATTTTCTGGCTCTGGCAGCG
 ACCATAGACGTCTTCGGGCCGGTCAGAGGAGTCGACGACTAGATGTGGGACTCGATGTCTCGGTCGCCGCACGGGCTATCTAAAAGACCGAGACCGTCGC
 TrpTyrLeuGlnLysProGlyGlnSerProGlnLeuLeuIleTyrThrLeuSerTyrArgAlaSerGlyValProAspArgPheSerGlySerGlySer>
```

```
  710       720       730       740       750       760       770       780       790       800
 GCACCGACTTCACCCTGAAGATCTCTAGAGTGGAAGCCGAGGACGTGGGCGTGTACTATTGCATGCAGCGGATCGAGTTCCCTCTGACCTTTGGCGGCGG
 CGTGGCTGAAGTGGGACTTCTAGAGATCTCACCTTCGGCTCCTGCACCCGCACATGATAACGTACGTCGCCTAGCTCAAGGGAGACTGGAAACCGCCGCC
GlyThrAspPheThrLeuLysIleSerArgValGluAlaGluAspValGlyValTyrTyrCysMetGlnArgIleGluPheProLeuThrPheGlyGlyGly>

  810       820       830       840       850       860       870       880       890       900
 AACAAAGGTGGAAATCAAGAAGAAGAAGCCCCTGGACGGCGAGTACTTCACACTGCAGATCCGGGGCAGAGAACGCTTCGAGATGTTCAGAGAGCTGAAC
 TTGTTTCCACCTTTAGTTCTTCTTCTTCGGGGACCTGCCGCTCATGAAGTGTGACGTCTAGGCCCCGTCTCTTGCGAAGCTCTACAAGTCTCTCGACTTG
  ThrLysValGluIleLysLysLysLysProLeuAspGlyGluTyrPheThrLeuGlnIleArgGlyArgGluArgPheGluMetPheArgGluLeuAsn>

  910       920       930       940       950       960
 GAGGCCCTGGAACTGAAGGATGCCCAGGCCGGAAAAGAGCCTGGACACCACCATCACCATCACT
 CTCCGGGACCTTGACTTCCTACGGGTCCGGCCTTTTCTCGGACCTGTGGTGGTAGTGGTAGTGA
 GluAlaLeuGluLeuLysAspAlaGlnAlaGlyLysGluProGlyHisHisHisHisHisHis>
```

B38-Fab-TD (VH-CH1-TD + VL-CL)

B38 VH-CH1-TD

```
   10        20        30        40        50        60        70        80        90       100
 ATGGGCTGGTCCTGCATCATCCTGTTTCTGGTGGCCACAGCCACAGGCGTGCACTCTGAAGTGCAGCTGGTTGAGTCTGGCGGAGGACTGGTTCAACCTG
 TACCCGACCAGGACGTAGTAGGACAAAGACCACCGGTGTCGGTGTCCGCACGTGAGACTTCACGTCGACCAACTCAGACCGCCTCCTGACCAAGTTGGAC
 MetGlyTrpSerCysIleIleLeuPheLeuValAlaThrAlaThrGlyValHisSerGluValGlnLeuValGluSerGlyGlyGlyLeuValGlnPro>

  110       120       130       140       150       160       170       180       190       200
 GCGGAAGCCTGAGACTGTCTTGTGCCGCCTCTGGCTTCATCGTGTCCAGCAACTACATGAGCTGGGTCCGACAGGCCCCTGGCAAAGGACTTGAATGGGT
 CGCCTTCGGACTCTGACAGAACACGGCGGAGACCGAAGTAGCACAGGTCGTTGATGTACTCGACCCAGGCTGTCCGGGGACCGTTTCCTGAACTTACCCA
GlyGlySerLeuArgLeuSerCysAlaAlaSerGlyPheIleValSerSerAsnTyrMetSerTrpValArgGlnAlaProGlyLysGlyLeuGluTrpVal>

  210       220       230       240       250       260       270       280       290       300
 GTCCGTGATCTACAGCGGCGGCAGCACCTACTACGCCGATTCTGTGAAGGGCAGATTCACCATCAGCCGGCACAACAGCAAGAACACCCTGTACCTGCAG
 CAGGCACTAGATGTCGCCGCCGTCGTGGATGATGCGGCTAAGACACTTCCCGTCTAAGTGGTAGTCGGCCGTGTTGTCGTTCTTGTGGGACATGGACGTC
  SerValIleTyrSerGlyGlySerThrTyrTyrAlaAspSerValLysGlyArgPheThrIleSerArgHisAsnSerLysAsnThrLeuTyrLeuGln>

  310       320       330       340       350       360       370       380       390       400
 ATGAACAGCCTGAGAGCCGAGGACACCGCCGTGTACTACTGTGCCAGAGAAGCCTACGGCATGGACGTGTGGGGACAGGGAACAACCGTGACAGTGTCTA
 TACTTGTCGGACTCTCGGCTCCTGTGGCGGCACATGATGACACGGTCTCTTCGGATGCCGTACCTGCACACCCCTGTCCCTTGTTGGCACTGTCACAGAT
 MetAsnSerLeuArgAlaGluAspThrAlaValTyrTyrCysAlaArgGluAlaTyrGlyMetAspValTrpGlyGlnGlyThrThrValThrValSer>

  410       420       430       440       450       460       470       480       490       500
 GCGCCAGCACAAAGGGCCCTAGCGTTTTCCCACTGGCTCCCAGCAGCAAGTCTACCTCTGGTGGAACAGCCGCTCTGGGCTGCCTGGTCAAGGATTACTT
 CGCGGTCGTGTTTCCCGGGATCGCAAAAGGGTGACCGAGGGTCGTCGTTCAGATGGAGACCACCTTGTCGGCGAGACCCGACGGACCAGTTCCTAATGAA
SerAlaSerThrLysGlyProSerValPheProLeuAlaProSerSerLysSerThrSerGlyGlyThrAlaAlaLeuGlyCysLeuValLysAspTyrPhe>

  510       520       530       540       550       560       570       580       590       600
 TCCCGAGCCTGTGACCGTGTCCTGGAATTCTGGCGCTCTGACAAGCGGCGTGCACACCTTTCCAGCTGTGCTGCAAAGCAGCGGCCTGTACTCTCTGAGC
 AGGGCTCGGACACTGGCACAGGACCTTAAGACCGCGAGACTGTTCGCCGCACGTGTGGAAAGGTCGACACGACGTTTCGTCGCCGGACATGAGAGACTCG
  ProGluProValThrValSerTrpAsnSerGlyAlaLeuThrSerGlyValHisThrPheProAlaValLeuGlnSerSerGlyLeuTyrSerLeuSer>

  610       620       630       640       650       660       670       680       690       700
 AGCGTGGTCACAGTGCCTAGCTCTAGCCTGGGCACCCAGACCTACATCTGCAATGTGAACCACAAGCCTAGCAACACCAAGGTGGACAAGAAGGTGGAAC
 TCGCACCAGTGTCACGGATCGAGATCGGACCCGTGGGTCTGGATGTAGACGTTACACTTGGTGTTCGGATCGTTGTGGTTCCACCTGTTCTTCCACCTTG
 SerValValThrValProSerSerSerLeuGlyThrGlnThrTyrIleCysAsnValAsnHisLysProSerAsnThrLysValAspLysLysValGlu>
  710       720       730       740       750       760       770       780       790       800
 CCAAGAGCTGCGACAAGACCCACACCAAGAAGAAGCCTCTGGACGGCGAGTACTTCACTCTGCAGATCCGGGGCAGAGAACGCTTCGAGATGTTCAGAGA
 GGTTCTCGACGCTGTTCTGGGTGTGGTTCTTCTTCGGAGACCTGCCGCTCATGAAGTGAGACGTCTAGGCCCCGTCTCTTGCGAAGCTCTACAAGTCTCT
ProLysSerCysAspLysThrHisThrLysLysLysProLeuAspGlyGluTyrPheThrLeuGlnIleArgGlyArgGluArgPheGluMetPheArgGlu>

  810       820       830       840       850       860       870
 GCTGAACGAGGCCCTGGAACTGAAGGATGCCCAGGCCGGAAAAGAGCCTGGACACCACCATCACCATCACTG
 CGACTTGCTCCGGGACCTTGACTTCCTACGGGTCCGGCCTTTTCTCGGACCTGTGGTGGTAGTGGTAGTGAC
  LeuAsnGluAlaLeuGluLeuLysAspAlaGlnAlaGlyLysGluProGlyHisHisHisHisHisHis>
```

B38 VL-CL

```
        10        20        30        40        50        60        70        80        90       100
 AGCTTATGGGCTGGTCCTGCATCATCCTGTTTCTGGTGGCCACAGCCACAGGCGTGCACAGCGATATCGTGATGACCCAGTCTCCTAGCTTCCTGAGCGC
 TCGAATACCCGACCAGGACGTAGTAGGACAAAGACCACCGGTGTCGGTGTCCGCACGTGTCGCTATAGCACTACTGGGTCAGAGGATCGAAGGACTCGCG
      MetGlyTrpSerCysIleIleLeuPheLeuValAlaThrAlaThrGlyValHisSerAspIleValMetThrGlnSerProSerPheLeuSerAla>
       110       120       130       140       150       160       170       180       190       200
 CAGCGTGGGCGACAGAGTGACCATTACATGTAGAGCCAGCCAGGGCATCAGCAGCTACCTGGCTTGGTATCAGCAGAAGCCTGGCAAGGCCCCTAAACTG
 GTCGCACCCGCTGTCTCACTGGTAATGTACATCTCGGTCGGTCCCGTAGTCGTCGATGGACCGAACCATAGTCGTCTTCGGACCGTTCCGGGGATTTGAC
  SerValGlyAspArgValThrIleThrCysArgAlaSerGlnGlyIleSerSerTyrLeuAlaTrpTyrGlnGlnLysProGlyLysAlaProLysLeu>
       210       220       230       240       250       260       270       280       290       300
 CTGATCTACGCCGCCAGCACACTGCAGTCTGGCGTGCCATCTAGATTTTCCGGCAGCGGCTCTGGCACCGAGTTCACCCTGACCATATCTAGCCTGCAGC
 GACTAGATGCGGCGGTCGTGTGACGTCAGACCGCACGGTAGATCTAAAAGGCCGTCGCCGAGACCGTGGCTCAAGTGGGACTGGTATAGATCGGACGTCG
 LeuIleTyrAlaAlaSerThrLeuGlnSerGlyValProSerArgPheSerGlySerGlySerGlyThrGluPheThrLeuThrIleSerSerLeuGln>

       310       320       330       340       350       360       370       380       390       400
 CTGAGGACTTCGCCACCTACTACTGCCAGCAGCTGAACAGCTACCCTCCTTACACCTTTGGCCAGGGCACCAAGCTGGAAATCAAGCGGACAGTGGCCGC
 GACTCCTGAAGCGGTGGATGATGACGGTCGTCGACTTGTCGATGGGAGGAATGTGGAAACCGGTCCCGTGGTTCGACCTTTAGTTCGCCTGTCACCGGCG
ProGluAspPheAlaThrTyrTyrCysGlnGlnLeuAsnSerTyrProProTyrThrPheGlyGlnGlyThrLysLeuGluIleLysArgThrValAlaAla>

       410       420       430       440       450       460       470       480       490       500
 TCCTAGCGTGTTCATCTTTCCACCTAGCGACGAACAGCTGAAGTCCGGCACAGCCTCTGTCGTGTGCCTGCTGAACAACTTCTACCCCAGAGAAGCCAAG
 AGGATCGCACAAGTAGAAAGGTGGATCGCTGCTTGTCGACTTCAGGCCGTGTCGGAGACAGCACACGGACGACTTGTTGAAGATGGGGTCTCTTCGGTTC
  ProSerValPheIlePheProProSerAspGluGlnLeuLysSerGlyThrAlaSerValValCysLeuLeuAsnAsnPheTyrProArgGluAlaLys>
```

```
       510        520        530        540        550        560        570        580        590        600
 GTGCAGTGGAAGGTGGACAACGCTCTGCAGTCCGGCAACTCCCAAGAGAGCGTGACAGAGCAGGACAGCAAGGACTCCACCTACAGCCTGAGCAGCACCC
 CACGTCACCTTCCACCTGTTGCGAGACGTCAGGCCGTTGAGGGTTCTCTCGCACTGTCTCGTCCTGTCGTTCCTGAGGTGGATGTCGGACTCGTCGTGGG
 ValGlnTrpLysValAspAsnAlaLeuGlnSerGlyAsnSerGlnGluSerValThrGluGlnAspSerLysAspSerThrTyrSerLeuSerSerThr>

        610        620        630        640        650        660        670        680        690        700
 TGACACTGAGCAAGGCCGACTACGAGAAGCACAAAGTGTACGCCTGCGAAGTGACCCACCAGGGCCTTTCTAGCCCTGTGACCAAGAGCTTCAACCGGGG
 ACTGTGACTCGTTCCGGCTGATGCTCTTCGTGTTTCACATGCGGACGCTTCACTGGGTGGTCCCGGAAAGATCGGGACACTGGTTCTCGAAGTTGGCCCC
LeuThrLeuSerLysAlaAspTyrGluLysHisLysValTyrAlaCysGluValThrHisGlnGlyLeuSerSerProValThrLysSerPheAsnArgGly>
 CGAATGTTG
 GCTTACAAC
  GluCys>
```

SARS-CoV2-S1

```
30        40        50        60        70        80        90        100       110       120
 ATGGGATGGTCTTGTATAATTCTGTTCCTGGTGGCAACAGCAACAGGAGTGCATAGCGTGAACCTGACCACCAGAACACAGCTGCCTCCAGCCTACACCA
 TACCCTACCAGAACATATTAAGACAAGGACCACCGTTGTCGTTGTCCTCACGTATCGCACTTGGACTGGTGGTCTTGTGTCGACGGAGGTCGGATGTGGT
 MetGlyTrpSerCysIleIleLeuPheLeuValAlaThrAlaThrGlyValHisSerValAsnLeuThrThrArgThrGlnLeuProProAlaTyrThr>

130       140       150       160       170       180       190       200       210       220
 ACAGCTTCACCAGAGGCGTGTACTACCCCGACAAGGTGTTCAGATCCAGCGTGCTGCACTCTACCCAGGACCTGTTCCTGCCTTTCTTCAGCAACGTGAC
 TGTCGAAGTGGTCTCCGCACATGATGGGGCTGTTCCACAAGTCTAGGTCGCACGACGTGAGATGGGTCCTGGACAAGGACGGAAAGAAGTCGTTGCACTG
AsnSerPheThrArgGlyValTyrTyrProAspLysValPheArgSerSerValLeuHisSerThrGlnAspLeuPheLeuProPhePheSerAsnValThr>

230       240       250       260       270       280       290       300       310       320
 CTGGTTCCACGCCATCCACGTGTCCGGCACCAATGGCACCAAGAGATTCGACAACCCCGTGCTGCCCTTCAACGACGGGGTGTACTTTGCCAGCACCGAG
 GACCAAGGTGCGGTAGGTGCACAGGCCGTGGTTACCGTGGTTCTCTAAGCTGTTGGGGCACGACGGGAAGTTGCTGCCCCACATGAAACGGTCGTGGCTC
  TrpPheHisAlaIleHisValSerGlyThrAsnGlyThrLysArgPheAspAsnProValLeuProPheAsnAspGlyValTyrPheAlaSerThrGlu>

330       340       350       360       370       380       390       400       410       420
 AAGTCCAACATCATCAGAGGCTGGATCTTCGGCACCACACTGGACAGCAAGACCCAGAGCCTGCTGATCGTGAACAACGCCACCAACGTGGTCATCAAAG
 TTCAGGTTGTAGTAGTCTCCGACCTAGAAGCCGTGGTGTGACCTGTCGTTCTGGGTCTCGGACGACTAGCACTTGTTGCGGTGGTTGCACCAGTAGTTTC
 LysSerAsnIleIleArgGlyTrpIlePheGlyThrThrLeuAspSerLysThrGlnSerLeuLeuIleValAsnAsnAlaThrAsnValValIleLys>

430       440       450       460       470       480       490       500       510       520
 TGTGCGAGTTCCAGTTCTGCAACGACCCCTTCCTGGGCGTCTACTACCACAAGAACAACAAGAGCTGGATGGAAAGCGAGTTCCGGGTGTACAGCAGCGC
 ACACGCTCAAGGTCAAGACGTTGCTGGGGAAGGACCCGCAGATGATGGTGTTCTTGTTGTTCTCGACCTACCTTTCGCTCAAGGCCCACATGTCGTCGCG
ValCysGluPheGlnPheCysAsnAspProPheLeuGlyValTyrTyrHisLysAsnAsnLysSerTrpMetGluSerGluPheArgValTyrSerSerAla>

530       540       550       560       570       580       590       600       610       620
 CAACAACTGCACCTTCGAGTACGTGTCCCAGCCTTTCCTGATGGACCTGGAAGGCAAGCAGGGCAACTTCAAGAACCTGCGCGAGTTCGTGTTCAAGAAC
 GTTGTTGACGTGGAAGCTCATGCACAGGGTCGGAAAGGACTACCTGGACCTTCCGTTCGTCCCGTTGAAGTTCTTGGACGCGCTCAAGCACAAGTTCTTG
  AsnAsnCysThrPheGluTyrValSerGlnProPheLeuMetAspLeuGluGlyLysGlnGlyAsnPheLysAsnLeuArgGluPheValPheLysAsn>

630       640       650       660       670       680       690       700       710       720
 ATCGACGGCTACTTCAAGATCTACAGCAAGCACACCCCTATCAACCTCGTGCGGGATCTGCCTCAGGGCTTCTCTGCTCTGGAACCCCTGGTGGATCTGC
 TAGCTGCCGATGAAGTTCTAGATGTCGTTCGTGTGGGGATAGTTGGAGCACGCCCTAGACGGAGTCCCGAAGAGACGAGACCTTGGGGACCACCTAGACG
 IleAspGlyTyrPheLysIleTyrSerLysHisThrProIleAsnLeuValArgAspLeuProGlnGlyPheSerAlaLeuGluProLeuValAspLeu>

730       740       750       760       770       780       790       800       810       820
 CCATCGGCATCAACATCACCCGGTTTCAGACACTGCTGGCCCTGCACAGAAGCTACCTGACACCTGGCGATAGCAGCTCTGGATGGACAGCTGGCGCCGC
 GGTAGCCGTAGTTGTAGTGGGCCAAAGTCTGTGACGACCGGGACGTGTCTTCGATGGACTGTGGACCGCTATCGTCGAGACCTACCTGTCGACCGCGGCG
ProIleGlyIleAsnIleThrArgPheGlnThrLeuLeuAlaLeuHisArgSerTyrLeuThrProGlyAspSerSerSerGlyTrpThrAlaGlyAlaAla>

830       840       850       860       870       880       890       900       910       920
 TGCCTACTATGTGGGATACCTGCAGCCTCGGACCTTCCTGCTGAAGTACAACGAGAACGGCACCATCACCGACGCCGTGGATTGTGCTCTGGATCCTCTG
 ACGGATGATACACCCTATGGACGTCGGAGCCTGGAAGGACGACTTCATGTTGCTCTTGCCGTGGTAGTGGCTGCGGCACCTAACACGAGACCTAGGAGAC
  AlaTyrTyrValGlyTyrLeuGlnProArgThrPheLeuLeuLysTyrAsnGluAsnGlyThrIleThrAspAlaValAspCysAlaLeuAspProLeu>

930       940       950       960       970       980       990       1000      1010      1020
 AGCGAGACAAAGTGCACCCTGAAGTCCTTCACCGTGGAAAAGGGCATCTACCAGACCAGCAACTTCCGGGTGCAGCCCACCGAATCCATCGTGCGGTTCC
 TCGCTCTGTTTCACGTGGGACTTCAGGAAGTGGCACCTTTTCCCGTAGATGGTCTGGTCGTTGAAGGCCCACGTCGGGTGGCTTAGGTAGCACGCCAAGG
 SerGluThrLysCysThrLeuLysSerPheThrValGluLysGlyIleTyrGlnThrSerAsnPheArgValGlnProThrGluSerIleValArgPhe>

030      1040      1050      1060      1070      1080      1090      1100      1110      1120
 CCAATATCACCAATCTGTGCCCCTTCGGCGAGGTGTTCAATGCCACCAGATTCGCCTCTGTGTACGCCTGGAACCGGAAGCGGATCAGCAATTGCGTGGC
 GGTTATAGTGGTTAGACACGGGGAAGCCGCTCCACAAGTTACGGTGGTCTAAGCGGAGACACATGCGGACCTTGGCCTTCGCCTAGTCGTTAACGCACCG
ProAsnIleThrAsnLeuCysProPheGlyGluValPheAsnAlaThrArgPheAlaSerValTyrAlaTrpAsnArgLysArgIleSerAsnCysValAla>

130      1140      1150      1160      1170      1180      1190      1200      1210      1220
 CGACTACTCCGTGCTGTACAACTCCGCCAGCTTCAGCACCTTCAAGTGCTACGGCGTGTCCCCTACCAAGCTGAACGACCTGTGCTTCACAAACGTGTAC
 GCTGATGAGGCACGACATGTTGAGGCGGTCGAAGTCGTGGAAGTTCACGATGCCGCACAGGGGATGGTTCGACTTGCTGGACACGAAGTGTTTGCACATG
  AspTyrSerValLeuTyrAsnSerAlaSerPheSerThrPheLysCysTyrGlyValSerProThrLysLeuAsnAspLeuCysPheThrAsnValTyr>

230      1240      1250      1260      1270      1280      1290      1300      1310      1320
 GCCGACAGCTTCGTGATCCGGGGAGATGAAGTGCGGCAGATTGCCCCTGGACAGACAGGCAAGATCGCCGACTACAACTACAAGCTGCCCGACGACTTCA
 CGGCTGTCGAAGCACTAGGCCCCTCTACTTCACGCCGTCTAACGGGGACCTGTCTGTCCGTTCTAGCGGCTGATGTTGATGTTCGACGGGCTGCTGAAGT
 AlaAspSerPheValIleArgGlyAspGluValArgGlnIleAlaProGlyGlnThrGlyLysIleAlaAspTyrAsnTyrLysLeuProAspAspPhe>

330      1340      1350      1360      1370      1380      1390      1400      1410      1420
 CCGGCTGTGTGATTGCCTGGAACAGCAACAACCTGGACTCCAAAGTCGGCGGCAACTACAATTACCTGTACCGGCTGTTCCGGAAGTCCAATCTGAAGCC
 GGCCGACACACTAACGGACCTTGTCGTTGTTGGACCTGAGGTTTCAGCCGCCGTTGATGTTAATGGACATGGCCGACAAGGCCTTCAGGTTAGACTTCGG
ThrGlyCysValIleAlaTrpAsnSerAsnAsnLeuAspSerLysValGlyGlyAsnTyrAsnTyrLeuTyrArgLeuPheArgLysSerAsnLeuLysPro>

430      1440      1450      1460      1470      1480      1490      1500      1510      1520
 CTTCGAGCGGGACATCTCCACCGAGATCTATCAGGCCGGCAGCACCCCTTGTAACGGCGTGGAAGGCTTCAACTGCTACTTCCCACTGCAGTCCTACGGC
 GAAGCTCGCCCTGTAGAGGTGGCTCTAGATAGTCCGGCCGTCGTGGGGAACATTGCCGCACCTTCCGAAGTTGACGATGAAGGGTGACGTCAGGATGCCG
  PheGluArgAspIleSerThrGluIleTyrGlnAlaGlySerThrProCysAsnGlyValGluGlyPheAsnCysTyrPheProLeuGlnSerTyrGly>
```

```
530       1540      1550      1560      1570      1580      1590      1600      1610      1620
  TTTCAGCCCACAAATGGCGTGGGCTACCAGCCTTACAGAGTGGTGGTGCTGAGCTTCGAGCTGCTGCATGCTCCTGCCACAGTGTGCGGCCCTAAGAAAA
  AAAGTCGGGTGTTTACCGCACCCGATGGTCGGAATGTCTCACCACCACGACTCGAAGCTCGACGACGTACGAGGACGGTGTCACACGCCGGGATTCTTTT
  PheGlnProThrAsnGlyValGlyTyrGlnProTyrArgValValValLeuSerPheGluLeuLeuHisAlaProAlaThrValCysGlyProLysLys>

630       1640      1650      1660      1670      1680      1690      1700      1710      1720
  GCACCAATCTCGTGAAGAACAAATGCGTGAACTTCAACTTCAACGGCCTGACCGGCACCGGCGTGCTGACAGAGAGCAACAAGAAGTTCCTGCCATTCCA
  CGTGGTTAGAGCACTTCTTGTTTACGCACTTGAAGTTGAAGTTGCCGGACTGGCCGTGGCCGCACGACTGTCTCTCGTTGTTCTTCAAGGACGGTAAGGT
 SerThrAsnLeuValLysAsnLysCysValAsnPheAsnPheAsnGlyLeuThrGlyThrGlyValLeuThrGluSerAsnLysLysPheLeuProPheGln>

730       1740      1750      1760      1770      1780      1790      1800      1810      1820
  GCAGTTCGGCCGGGATATCGCCGATACCACAGATGCCGTCAGAGATCCCCAGACACTGGAAATCCTGGACATCACCCCTTGCAGCTTCGGCGGAGTGTCT
  CGTCAAGCCGGCCCTATAGCGGCTATGGTGTCTACGGCAGTCTCTAGGGGTCTGTGACCTTTAGGACCTGTAGTGGGGAACGTCGAAGCCGCCTCACAGA
   GlnPheGlyArgAspIleAlaAspThrThrAspAlaValArgAspProGlnThrLeuGluIleLeuAspIleThrProCysSerPheGlyGlyValSer>

830       1840      1850      1860      1870      1880      1890      1900      1910      1920
  GTGATCACCCCTGGCACCAACACCAGCAATCAGGTGGCAGTGCTGTACCAGGACGTGAACTGTACCGAAGTGCCCGTGGCCATTCACGCCGATCAGCTGA
  CACTAGTGGGGACCGTGGTTGTGGTCGTTAGTCCACCGTCACGACATGGTCCTGCACTTGACATGGCTTCACGGGCACCGGTAAGTGCGGCTAGTCGACT
  ValIleThrProGlyThrAsnThrSerAsnGlnValAlaValLeuTyrGlnAspValAsnCysThrGluValProValAlaIleHisAlaAspGlnLeu>

930       1940      1950      1960      1970      1980      1990      2000      2010      2020
  CACCTACATGGCGGGTGTACTCCACCGGCAGCAATGTGTTTCAGACCAGAGCCGGCTGTCTGATCGGAGCCGAGCACGTGAACAATAGCTACGAGTGCGA
  GTGGATGTACCGCCCACATGAGGTGGCCGTCGTTACACAAAGTCTGGTCTCGGCCGACAGACTAGCCTCGGCTCGTGCACTTGTTATCGATGCTCACGCT
 ThrProThrTrpArgValTyrSerThrGlySerAsnValPheGlnThrArgAlaGlyCysLeuIleGlyAlaGluHisValAsnAsnSerTyrGluCysAsp>

030       2040      2050      2060      2070      2080      2090      2100      2110      2120
  CATCCCCATCGGCGCTGGCATCTGCGCCTCTTACCAGACACAGACAAACAGCCCCAGACGGGCCAGATCTGTGGCTTCTCAGGGCCTGAACGACATCTTC
  GTAGGGGTAGCCGCGACCGTAGACGCGGAGAATGGTCTGTGTCTGTTTGTCGGGGTCTGCCCGGTCTAGACACCGAAGAGTCCCGGACTTGCTGTAGAAG
   IleProIleGlyAlaGlyIleCysAlaSerTyrGlnThrGlnThrAsnSerProArgArgAlaArgSerValAlaSerGlnGlyLeuAsnAspIlePhe>

130       2140      2150      2160      2170      2180
  GAGGCTCAGAAAATCGAATGGCACGAGCACCACCACCATCACCACTGAGCG
  CTCCGAGTCTTTTAGCTTACCGTGCTCGTGGTGGTGGTAGTGGTGACTCGC
  GluAlaGlnLysIleGluTrpHisGluHisHisHisHisHisHis***>
```

SARS-CoV2-RBD

```
     10        20        30        40        50        60        70        80        90        100
  ATGGGATGGTCTTGTATAATTCTGTTCCTGGTGGCAACAGCAACAGGAGTGCATAGCAATATCACCAATCTGTGCCCCTTCGGCGAGGTGTTCAATGCCA
  TACCCTACCAGAACATATTAAGACAAGGACCACCGTTGTCGTTGTCCTCACGTATCGTTATAGTGGTTAGACACGGGGAAGCCGCTCCACAAGTTACGGT
  MetGlyTrpSerCysIleIleLeuPheLeuValAlaThrAlaThrGlyValHisSerAsnIleThrAsnLeuCysProPheGlyGluValPheAsnAla>

    110       120       130       140       150       160       170       180       190       200
  CCAGATTCGCCTCTGTGTACGCCTGGAACCGGAAGCGGATCAGCAATTGCGTGGCCGACTACTCCGTGCTGTACAACTCCGCCAGCTTCAGCACCTTCAA
  GGTCTAAGCGGAGACACATGCGGACCTTGGCCTTCGCCTAGTCGTTAACGCACCGGCTGATGAGGCACGACATGTTGAGGCGGTCGAAGTCGTGGAAGTT
 ThrArgPheAlaSerValTyrAlaTrpAsnArgLysArgIleSerAsnCysValAlaAspTyrSerValLeuTyrAsnSerAlaSerPheSerThrPheLys>

    210       220       230       240       250       260       270       280       290       300
  GTGCTACGGCGTGTCCCCTACCAAGCTGAACGACCTGTGCTTCACAAACGTGTACGCCGACAGCTTCGTGATCCGGGGAGATGAAGTGCGGCAGATTGCC
  CACGATGCCGCACAGGGGATGGTTCGACTTGCTGGACACGAAGTGTTTGCACATGCGGCTGTCGAAGCACTAGGCCCCTCTACTTCACGCCGTCTAACGG
   CysTyrGlyValSerProThrLysLeuAsnAspLeuCysPheThrAsnValTyrAlaAspSerPheValIleArgGlyAspGluValArgGlnIleAla>

    310       320       330       340       350       360       370       380       390       400
  CCTGGACAGACAGGCAAGATCGCCGACTACAACTACAAGCTGCCCGACGACTTCACCGGCTGTGTGATTGCCTGGAACAGCAACAACCTGGACTCCAAAG
  GGACCTGTCTGTCCGTTCTAGCGGCTGATGTTGATGTTCGACGGGCTGCTGAAGTGGCCGACACACTAACGGACCTTGTCGTTGTTGGACCTGAGGTTTC
  ProGlyGlnThrGlyLysIleAlaAspTyrAsnTyrLysLeuProAspAspPheThrGlyCysValIleAlaTrpAsnSerAsnAsnLeuAspSerLys>

    410       420       430       440       450       460       470       480       490       500
  TCGGCGGCAACTACAATTACCTGTACCGGCTGTTCCGGAAGTCCAATCTGAAGCCCTTCGAGCGGGACATCTCCACCGAGATCTATCAGGCCGGCAGCAC
  AGCCGCCGTTGATGTTAATGGACATGGCCGACAAGGCCTTCAGGTTAGACTTCGGGAAGCTCGCCCTGTAGAGGTGGCTCTAGATAGTCCGGCCGTCGTG
 ValGlyGlyAsnTyrAsnTyrLeuTyrArgLeuPheArgLysSerAsnLeuLysProPheGluArgAspIleSerThrGluIleTyrGlnAlaGlySerThr>

    510       520       530       540       550       560       570       580       590       600
  CCCTTGTAACGGCGTGGAAGGCTTCAACTGCTACTTCCCACTGCAGTCCTACGGCTTTCAGCCCACAAATGGCGTGGGCTACCAGCCTTACAGAGTGGTG
  GGGAACATTGCCGCACCTTCCGAAGTTGACGATGAAGGGTGACGTCAGGATGCCGAAAGTCGGGTGTTTACCGCACCCGATGGTCGGAATGTCTCACCAC
   ProCysAsnGlyValGluGlyPheAsnCysTyrPheProLeuGlnSerTyrGlyPheGlnProThrAsnGlyValGlyTyrGlnProTyrArgValVal>

    610       620       630       640       650       660
  GTGCTGAGCTTCGAGCTGCTGCATGCTCCTGCCACAGTGCACCACCACCATCACCAC
  CACGACTCGAAGCTCGACGACGTACGAGGACGGTGTCACGTGGTGGTGGTAGTGGTG
  ValLeuSerPheGluLeuLeuHisAlaProAlaThrValHisHisHisHisHisHis
```

Supplementary Figure 4

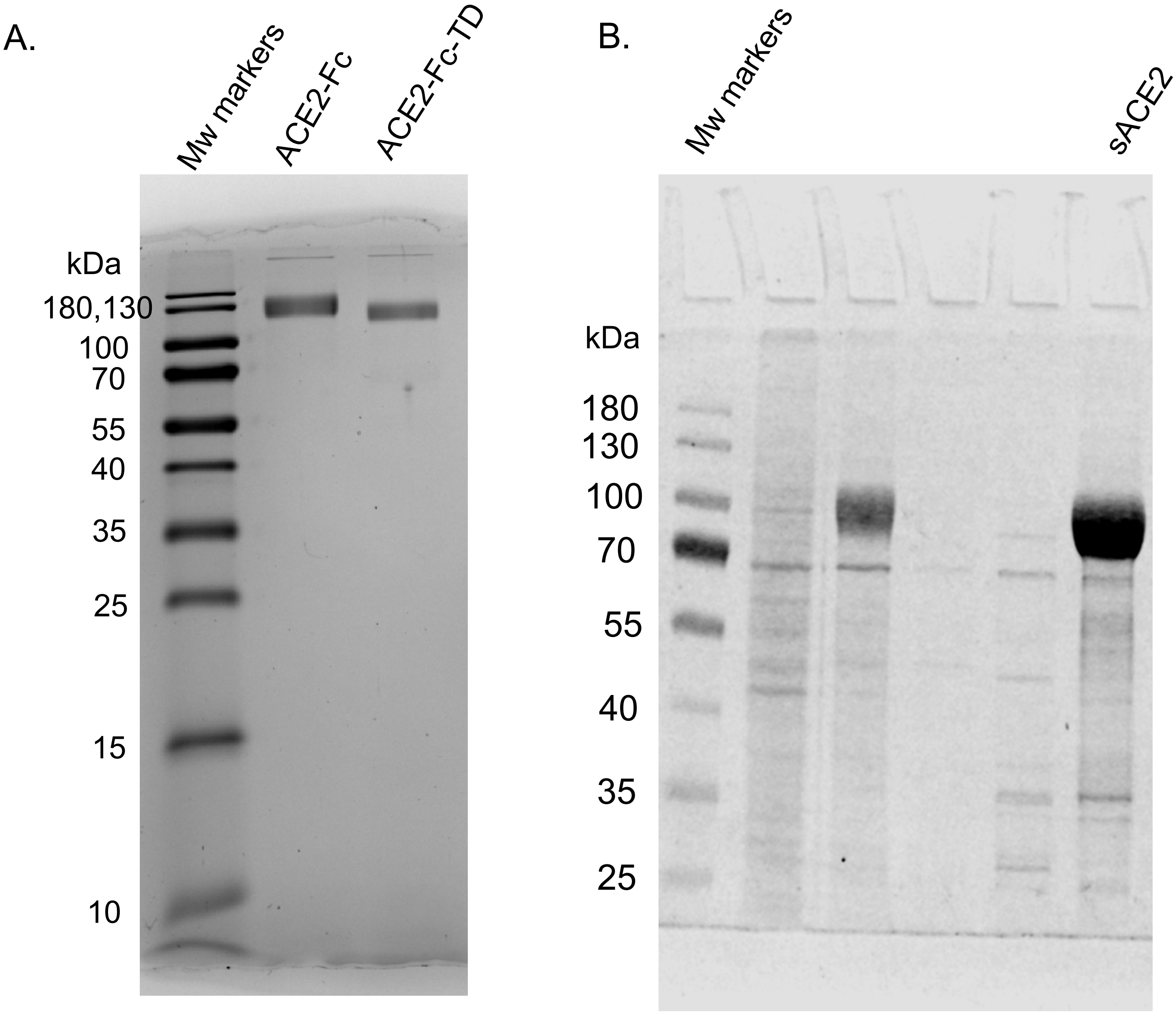

Supplementary Figure 4. Uncropped SDS-PAGE gel images shown in Figure 1A.

The images show full length Coomassie stained SDS-PAGE gels (with indicated stained molecular weight markers).
